# Commensal Leucothoidae (Crustacea, Amphipoda) of the Ryukyu Archipelago, Japan. Part I: ascidian-dwellers

**DOI:** 10.3897/zookeys.163.2003

**Published:** 2012-01-09

**Authors:** Kristine N. White, James Davis Reimer

**Affiliations:** 1 Rising Star Program, Trans-disciplinary Organization for Subtropical Island Studies (TRO-SIS), University of the Ryukyus, 1 Senbaru, Nishihara, Okinawa, Japan

**Keywords:** Leucothoidae, Ryukyu, Okinawa, Japan, new species, commensal, *Leucothoe amamiensis*, *Leucothoe elegans*, *Leucothoe nathani*, *Leucothoe obuchii*, *Leucothoe trulla*, *Leucothoe vulgaris*, *Paranamixis thomasi*

## Abstract

Commensal leucothoid amphipods have been collected from the branchial chambers of their ascidian hosts throughout the Ryukyu Archipelago, Japan. Seven new species are described in two genera with valuable location data and host records. An identification key to ascidian-dwelling Leucothoidae of the Ryukyu Archipelago is provided.

## Introduction

The Leucothoidae are a family of marine gammaridean amphipods that can be found inhabiting sessile invertebrate hosts worldwide. The family currently contains 139 species in five genera and can be divided into two clades (White 2011). The anamixid clade inhabits tropical to warm temperate waters and exhibits extreme sexual dimorphism. Terminal males are referred to as anamorphs and subterminal males and females are referred to as leucomorphs (Thomas and Barnard 1983). This clade contains the genera *Anamixis* Stebbing, 1897, *Nepanamixis* Thomas, 1997, and *Paranamixis* Schellenberg, 1938. The leucothoid clade inhabits tropical to polar waters and exhibits minimal to moderate sexual dimorphism. This clade contains the genera *Leucothoe* Leach, 1814 and *Paraleucothoe* Stebbing, 1899.

Leucothoids are typically found as endocommensal associates of sponges, ascidians or bivalve mollusks, where they utilize the feeding current produced by their hosts to feed. All genera exhibit extended parental care with members of the anamixid clade being potentially eusocial. Anamixids exhibit two of the three criteria for eusociality presented by Michener (1969). They have colonies with overlapping generations and an organized caste system with different morphologies. The third criterion, having reproduction restricted to certain individuals, has yet to be confirmed in the Leucothoidae (White 2010).

There are currently seven Leucothoidae species and one leucomorph reported from Japan (*Anamixis* sp. Hirayama, 1985 (leucomorph); *Leucothoe alata* (Barnard, 1959); *Leucothoe bidens* Hirayama, 1985; *Leucothoe nagatai* Ishimaru, 1985; *Leucothoe pacifica* Nagata, 1963; *Leucothoe stylifera* Stimpson, 1856; *Paranamixis aberro* Hirayama, 1983; *Paranamixis misakiensis* Thomas, 1997). The leucomorph is not connected to an anamorph and is, therefore, not considered a valid species (White 2011). All currently described Japanese leucothoids are reported from mainland Japan, with no species documented from the Ryukyu Archipelago. Of these seven species, only *Leucothoe nagatai* has been reported with its host. *Leucothoe stylifera* has never been illustrated; its type locality is simply Japan and it has no associated host or depth record. This lack of detailed host and locality records extends to leucothoid species reported worldwide.

**Figure 1.**
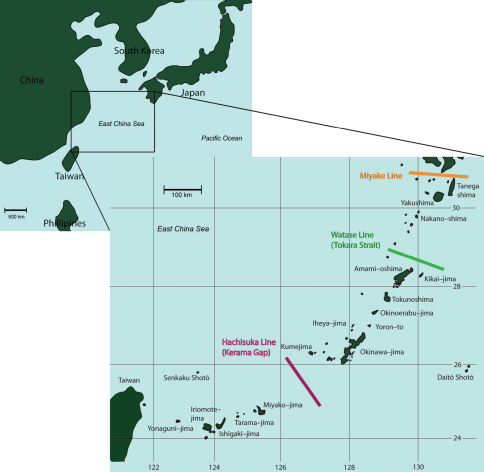
Map of the Ryukyu Archipelago, Japan.

The Ryukyu Archipelago consists of over 900 islands and islets between mainland Japan and Taiwan (Fig. 1) and is an area that has not been investigated for most amphipod families including leucothoids. The triple junction of the Philippine, Pacific, and Eurasian plates provides an interesting biogeographic study area, bringing together three potentially different sets of species. Roberts et al. (2002) states that Indo-Pacific reefs are the most diverse areas in the world with high levels of endemicity. The Ryukyu Archipelago is considered a center of endemism, perhaps due to the Kuroshio Current moving waters from tropical to temperate latitudes (Roberts et al. 2002; Hughes et al. 2002). There are currently recognized biogeographic boundaries within the Ryukyu Archipelago; the Hachisuka, Watase, and Miyake lines (Fig. 1). These boundaries apply to terrestrial organisms such as insects, mammals, reptiles, amphibians, and birds due to the past connection of the island chain to the Eurasian continent by land bridges (Kizaki and Oshiro 1977, 1980; Ota et al. 2004). Whether these boundaries apply to marine species is unknown. It is possible the boundaries may apply to marine species with restricted distributions, such as peracarid crustaceans that lack a dispersive pelagic larval stage.

## Methods

**Figure 2.**
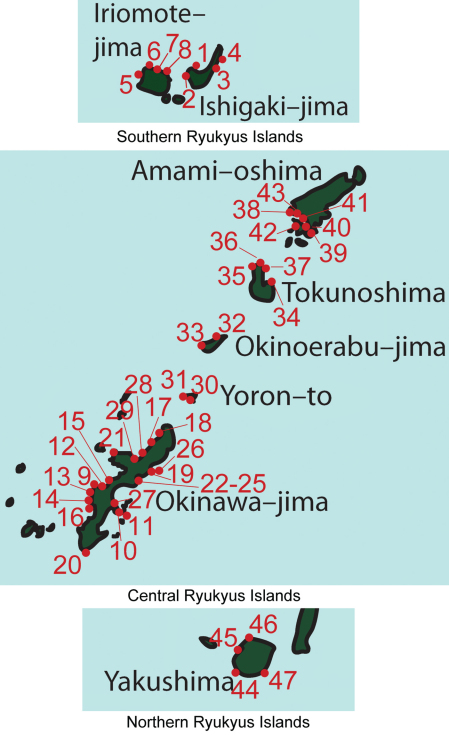
Map of collection localities in the Ryukyu Archipelago, Japan.

Specimens were collected via snorkeling and SCUBA at 47 locations throughout the Ryukyu Archipelago: Ishigaki–jima Island (4), Iriomote–jima Island (4), Okinawa–jima Island (21), Yoron–to Island (2), Okinoerabu–jima Island (2), Tokunoshima Island (4), Amami–oshima Island (6), and Yakushima Island (4) (Fig. 2). Table 1 lists the collection localities numbered in Fig. 2. Detailed station data are available in Supplementary Table 1. Whole ascidians were collected in zip-lock plastic bags and brought back to the laboratory. The ascidians were then dissected and commensal leucothoid amphipods were removed from the branchial chambers. Amphipods were preserved in 2% seawater buffered formalin for morphological analysis and 99% ethanol for molecular studies.

**Table 1.** Collection localities numbered in Fig. 2.

**Southern Ryukyu Islands**	
1	Kibura Bay, Ishigaki–jima Island, Okinawa
2	Sukoji Beach, Ishigaki–jima Island, Okinawa
3	Inoda Beach, Ishigaki–jima Island, Okinawa
4	Ibaruma, Ishigaki–jima Island, Okinawa
5	S of Hoshitate, Iriomote–jima Island, Okinawa
6	Channel between Iriomote–jima Island and Hatoma–jima Island, Okinawa
7	Blue Hole, Iriomote–jima Island, Okinawa
8	River drainage east of Funauki Port, Iriomote–jima Island, Okinawa
**Central Ryukyu Islands**	
9	Zanpa Cape, Okinawa–jima Island, Okinawa
10	Route 10 Bridge, Okinawa–jima Island, Okinawa
11	Hamahiga–jima Island, Okinawa
12	Maeda Point, Okinawa–jima Island, Okinawa
13	Toguchi Beach, Okinawa–jima Island, Okinawa
14	Mizugama, Okinawa–jima Island, Okinawa
15	Manza, Okinawa–jima Island, Okinawa
16	Sunabe Seawall, Okinawa–jima Island, Okinawa
17	Yona, Okinawa–jima Island, Okinawa
18	Zatsun, Okinawa–jima Island, Okinawa
19	Teniya Beach, Okinawa–jima Island, Okinawa
20	Odo, Okinawa–jima Island, Okinawa
21	Bise, Okinawa–jima Island, Okinawa
22	Chiribishi, Oura–wan Bay, Okinawa–jima Island, Okinawa
23	Kita–nakase, Oura–wan Bay, Okinawa–jima Island, Okinawa
24	Tettou–mae–oki, Oura–wan Bay, Okinawa–jima Island, Okinawa
25	Umi-saboten, Oura–wan Bay, Okinawa–jima Island, Okinawa
26	Teniya, Okinawa–jima Island, Okinawa
27	Uken, Kin Bay, Okinawa–jima Island, Okinawa
28	Shioya Bay, Okinawa–jima Island, Okinawa
29	Haneji Bay, Okinawa–jima Island, Okinawa
30	Ukatchi Coast, Yoron–to Island, Kagoshima
31	Shinaha Coast, Yoron–to Island, Kagoshima
32	Wanjo Beach, Okinoerabu–jima Island, Kagoshima
33	Naikina, Okinoerabu–jima Island, Kagoshima
34	Kaminomine, Tokunoshima Island, Kagoshima
35	Tete, Tokunoshima Island, Kagoshima
36	Omonawa, Tokunoshima Island, Kagoshima
37	San, Tokunoshima Island, Kagoshima
38	Shirahama Beach, Amami–oshima Island, Kagoshima
39	Kuse, Kakeroma–jima Island, Kagoshima
40	Sanakuiwa, Amami–oshima Island, Kagoshima
41	Boat dock, Amami–oshima Island, Kagoshima
42	Nominoura Oku, Kakeroma–jima Island, Kagoshima
43	Konase Kurosaki, Amami–oshima Island, Kagoshima
**Northern Ryukyu Islands**	
44	Kurio, Yakushima Island, Kagoshima
45	Yoshida, Yakushima Island, Kagoshima
46	Isso, Yakushima Island, Kagoshima
47	Haruta, Yakushima Island, Kagoshima

Other sampling efforts included isolating entire sponges in zip-lock plastic bags for dissection in the laboratory or capturing amphipods individually in situ, using a modified squirt bottle. Coral rubble samples were also taken, elutriated, and sieved on location using both saltwater and formalin washes. Samples were sorted immediately and amphipods were preserved as previously stated.

Specimens used for morphological analyses were transferred to glycerin, dissected, mounted on slides, and illustrated using a Nikon^® ^Y-IDT drawing tube attached to a Nikon^®^ Eclipse 50I compound microscope. Pencil drawings were scanned and digitally inked in Adobe^®^ Illustrator using a Wacom^®^ Tablet, following the methods of Coleman (2003).

Descriptions are of males with sexually dimorphic characters described in a separate section. Terminology used in descriptions follows White and Thomas (2009) with ‘proximal margin’ of the carpus and dactylus referring to the margins closing on the propodus. Setae nomenclature follows Oshel and Steele (1988) where possible without having SEM images for the specimens described here. All setae are simple, unless noted.

Type material is deposited in The University of the Ryukyus Museum (Fujukan), with the prefix RUMF for museum numbers. Additional material has been deposited in The National Museum of Nature and Science in Tokyo, with the prefix NSMT for museum numbers.

Scale bars in figures represent 0.1 mm unless noted.

Figure legend: Hd, head; Mx, maxilla; Md, mandible; Xpd, maxilliped; LL, lower lip; UL, upper lip; G, gnathopod; P, pereopod; T, telson; U, uropod; L, left; R, right; l, lateral; m, medial; +, enlarged.

## Taxonomy

### Leucothoe

Leach, 1814

http://species-id.net/wiki/Leucothoe

#### Generic diagnosis.

Eyes, if present, generally well developed with 10 or more ocelli. Mandibles lacking molars, palp three articulate; right lacinia mobilis smaller than left. Maxilliped inner plates fused, palp 4–articulate; outer plates not reaching apex of palp article 1. Coxa 1–4 relatively equal in widths. Pereopods 5–7 bases generally expanded. Minimal to no sexual dimorphism.

#### Leucothoe amamiensis sp. n.

urn:lsid:zoobank.org:act:33B0D200-A04A-4CA1-BFEA-5710AA382B05

http://species-id.net/wiki/Leucothoe_amamiensis

Figs 3 4

##### Type material.

Holotype male, 5.9 mm, RUMF-ZC-1654, Sanakuiwa, Amami–oshima Island patch reef (28°06'58"N, 129°22'01"E), in branchial chamber of solitary ascidians, *Pyura microcosmus* (Savigny, 1816), 10 m, K.N. White, col., 19 March 2011 (KNWAmami2H). Paratype female, 4.7 mm, RUMF-ZC-1655, same station data as holotype.

##### Type locality.

Sanakuiwa, Amami–oshima Island, Japan (28°06'58"N, 129°22'01"E).

##### Additional material examined. 

1 specimen, NSMT-Cr21813, KNWAmami2E; 3 specimens, RUMF-ZC-1656, KNWAmami2H; 10 specimens, RUMF-ZC-1657, KNWAmami3A; 33 specimens, NSMT-Cr21814, KNWAmami3C; 2 specimens, RUMF-ZC-1698, KNWAmami47J.

##### Diagnosis (male).

Mandibular palp article 2 with 17 setae. Right mandible lacinia mobilis with 2 distal rows of dentition. Coxae 1–4 with several short lateral facial setae. Gnathopod 1 coxa with 1 long medial facial seta. Gnathopod 2 basis anterior margin with 19 short and medium setae; carpus with large subdistal tooth; propodus mediofacial setal row displaced to palm. Epimeron 1 with anteroventral tuft of setae. Telson with plumose facial setae and simple marginal setae, apex truncate.

##### Description (male).

Head. Anterior margin rounded, anterodistal margin evenly rounded; ventral cephalic keel anterior margin excavate, anteroventral margin quadrate with a simple cusp, ventral margin oblique; eyes with more than 10 ommatidia, round. Antenna 1 0.3 × body length, flagellum 8–articulate, peduncle article 1 width less than 2 × article 2, accessory flagellum 1–articulate, aesthetascs present. Antenna 2 0.3 × body length, slightly shorter than antenna 1, flagellum 3–articulate. Mandibular palp ratio of articles 1–3 1.0: 3.5: 1.5, article 2 with 17 setae, article 3 with 2 distal setae, incisors strongly dentate; left mandible with 14 raker spines, lacinia mobilis large, strongly toothed; right mandible with 15 raker spines, lacinia mobilis small, weakly dentate, with 2 rows of dentition. Upper lip asymmetrically lobate, anterior margin setose. Lower lip inner lobes fused, bare; outer lobes with moderate gape, anterior margins setose. Maxilla 1 palp 2–articulate with 3 distal slender setae; outer plate with 7 distal robust setae and 3 distal slender setae. Maxilla 2 inner plate with 2 robust and 7 slender distal setae, short row of facial setae; outer plate with 11 distal marginal setae, facial setae present. Maxilliped inner plates distal margin with v-shaped indentation, with short robust setae; outer plate inner margin smooth, reaching 0.3 × palp article 1, with simple and setulate-serrate marginal setae, facial setae present; palp article 4 subequal in length with article 3, distally acute.

Pereon. Coxae 1–4 relative widths 1.0: 1.0: 0.7: 1.4. Gnathopod 1 coxa smooth, with tiny marginal setae, anterodistal margin produced, subquadrate, posterior margin excavate, medial and lateral facial setae present; basis distally expanded, anterior margin with 18 setae, posterior margin bare; ischium with 1 posterodistal seta; carpus linear, length 9.1 × width, proximal margin smooth, distal margin with 3 short setae; propodus straight, palm dentate with 6 proximal setae; dactylus smooth, reaching 0.3 × propodus length. Gnathopod 2 coxa longer than broad, subequal in length with coxa 3, smooth, with tiny marginal setae, anterodistally rounded, distal margin straight, posterior margin straight, lateral facial setae present; basis distally expanded with 3 small anterior tubercles, anterior margin with 19 short and medium setae, posterior margin with 2 short setae; ischium with posterior, distal, and posterodistal setae; carpus 0.3 × propodus length, curved, with large subdistal tooth, anterior margin smooth; propodus with 1 mediofacial setal row displaced to palm, reaching 0.8 × propodus length, with 1 row of submarginal setae, posterior margin smooth, palm convex with 3 small and 1 major tubercle, indentation near distal end of dactylus; dactylus curved, proximal margin smooth with 1 seta, anterior margin distally subacute, reaching 0.6 × propodus length. Pereopod 3 coxa length 1.8 × width, anterodistal corner overriding distal face of coxa 2, extending below it, smooth, with tiny marginal setae, anterior margin straight, distal margin oblique, posterior margin straight, lateral facial setae present. Pereopod 4 coxa smooth, with tiny marginal setae, anterior margin tapered, distal margin produced, posterior margin excavate, lateral facial setae present. Pereopods 5–7 coxae facial setae present; bases width length ratios 1: 1.4, 1: 1.3, 1: 1.3, posterior margins smooth, setose.

Pleon. Epimeron 1 with tuft of anteroventral setae, epimera 2–3 with ventral setae; epimeron 3 posteroventral corner subquadrate, produced. Uropods 1–3 relative lengths 1.0: 0.9: 1.1. Uropod 1 peduncle and outer ramus subequal in length with inner ramus; inner ramus with 6 robust setae; outer ramus with 2 robust setae. Uropod 2 peduncle 0.8 × inner ramus length; outer ramus 0.7 × inner ramus length; inner ramus with 5 robust setae; outer ramus with 3 robust setae. Uropod 3 peduncle 1.1 × inner ramus length; outer ramus subequal in length with inner ramus; inner and outer rami each with 3 robust setae. Telson 2.7 × longer than wide, with plumose facial setae and simple marginal setae, apex truncate.

##### Female (sexually dimorphic characters).

Gnathopod 1 basis proximally expanded, anterior margin with 13 short setae, posterior margin with 16 short posterior setae; ischium with posterior seta; carpus distal margin with 4 short setae; propodus palm with 5 proximal setae. Gnathopod 2 basis without tubercles, anterior margin with 26 short and long setae, posterior margin with 12 short setae; ischium with 14 posterior setae, 3 anterior setae, and 2 posterodistal setae; carpus with small subdistal tooth; propodus palm with small tubercles.

**Figure 3.**
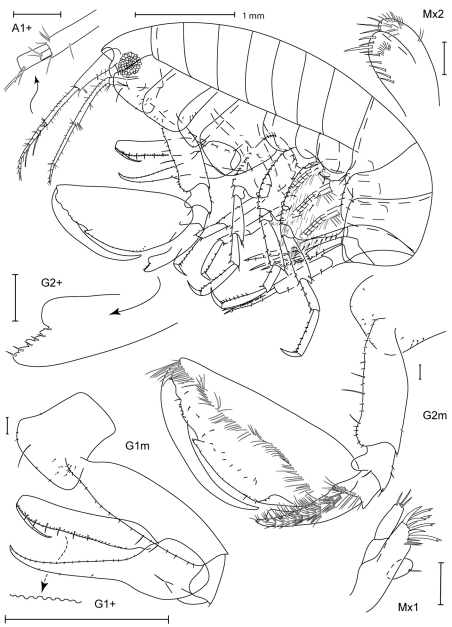
*Leucothoe amamiensis* sp. n., holotype male, 5.9 mm, RUMF-ZC-1654.

**Figure 4.**
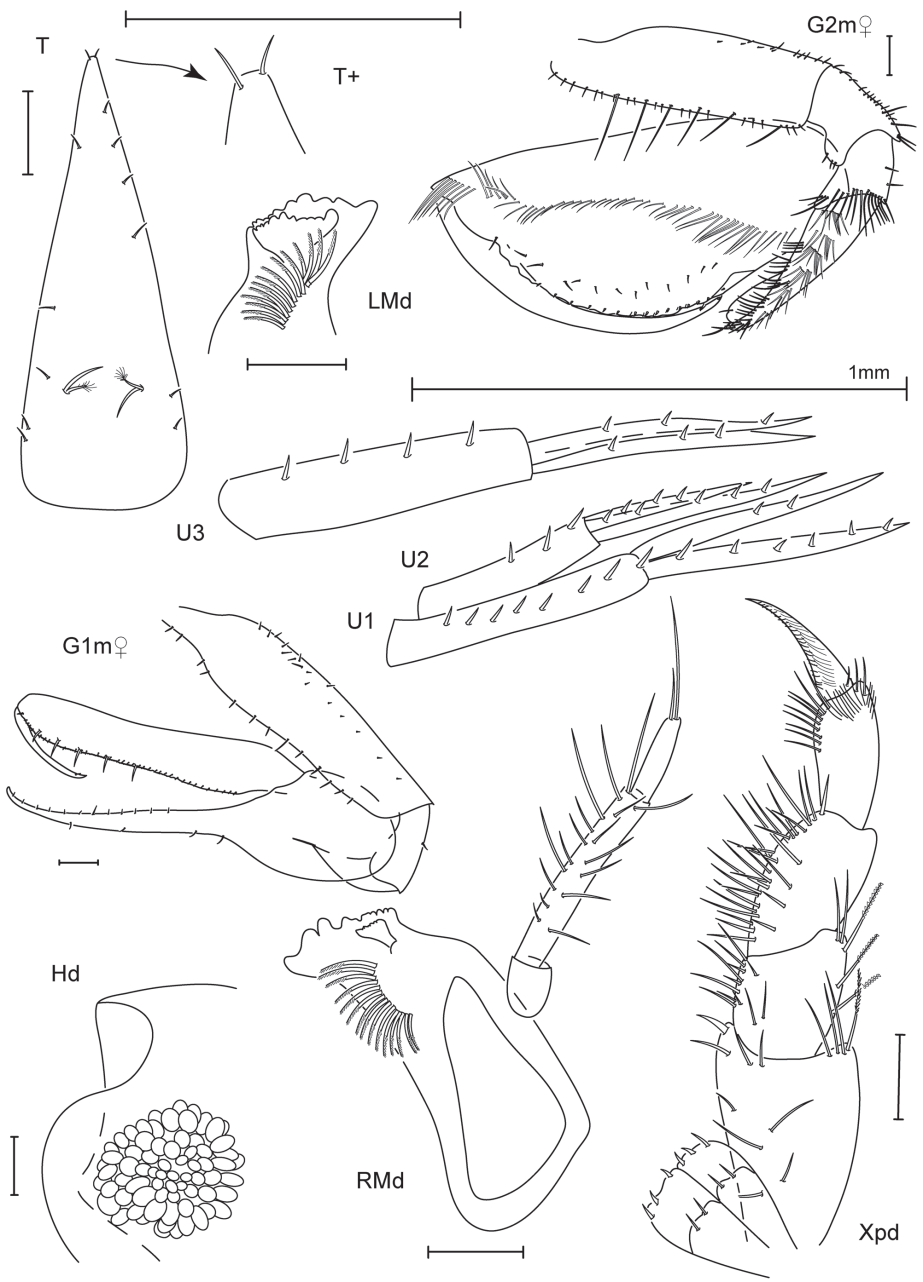
*Leucothoe amamiensis* sp. n., holotype male, 5.9 mm, RUMF-ZC-1654; paratype female, 4.7 mm, RUMF-ZC-1655.

##### Etymology.

After ‘Amami’ and referring to the type locality of this species.

##### Ecology.

In branchial chamber of solitary ascidians, *Rhopalaea circula* Monniot & Monniot, 2001 (Fig. 18F); *Pyura microcosmus* (Fig. 18D); and coral rubble.

##### Relationships.

*Leucothoe amamiensis* is similar to *Leucothoe commensalis* Haswell, 1879, *Leucothoe wuriti* Thomas and Klebba, 2007, *Leucothoe epidemos* White and Thomas, 2009, and *Leucothoe thula* White and Thomas, 2009 in having a rounded head, single seta on the medial surface of coxa 1, a long gnathopod 1 dactylus, and a displaced gnathopod 2 mediofacial setal row. It is similar to *Leucothoe articulosa* Montagu, 1804, *Leucothoe incisa* (Robertson, 1893), and *Leucothoe procera* (Bate, 1857) in having gnathopod 2 carpus with a large subapical tooth. *Leucothoe amamiensis* is similar to *Leucothoe occulta* Krapp-Schickel, 1975 in having coxae with facial setae. *Leucothoe amamiensis* differs from these species in having two rows of dentition on the right mandibular lacinia mobilis, a heavily setose gnathopod 2 basis, and a telson with plumose facial setae and simple marginal setae, and truncate apex.

##### Remarks.

*Leucothoe amamiensis* has a pink-orange striped color pattern (Fig. 17B). This species appears to be endemic to Amami–oshima Island.

##### Distribution.

East China Sea: Amami–oshima Island, Kagoshima, Japan.

#### Leucothoe elegans sp. n.

urn:lsid:zoobank.org:act:9113E46D-8259-463F-BBF9-9BBB2FAA5ED4

http://species-id.net/wiki/Leucothoe_elegans

Figs 5 6

##### Type material.

Holotype male, 4.5 mm, RUMF-ZC-1658, Isso, Yakushima Island patch reef (30°27'29"N, 130°29'22"E), in grey-purple hard sponge, 10–12 m, K.N. White, col., 27 May 2011 (KNWYaku3A). Paratype female, 5.7 mm, RUMF-ZC-1659, same station data as holotype.

**Type locality.** Isso, Yakushima Island, Japan (30°27'29"N, 130°29'22"E).

##### Additional Material Examined.

8 specimens, NSMT-Cr21815, KNWYaku3B; 4 specimens, RUMF-ZC-1660, KNWYaku3L; 3 specimens, RUMF-ZC-1661, KNWYaku5C; 3 specimens, RUMF-ZC-1662, KNWOkinawa54A; 16 specimens, NSMT- Cr 21816, KNWOkinawa54I; 2 specimens, RUMF-ZC-1699, KNWOkinawa54I.

**Diagnosis (male).** Mandibular palp article 2 robust, with 4 setae. Right mandible lacinia mobilis distal margin with 3 rows of dentition. Maxilliped outer plate inner margin tuberculate. Eye large, covering most head. Gnathopod 1 basis proximally widened; carpus elongate. Gnathopod 2 propodus with one submarginal row of robust setae; dactylus proximal margin with 2 setae, distal margin with spine; epimeron 3 posteroventral margin with small sinus.

##### Description (male).

Head. Anterior margin truncate, anterodistal margin evenly rounded; ventral cephalic keel anterior margin transverse, anteroventral margin rounded, ventral margin straight; eyes with more than 10 ommatidia, large, round. Antenna 1 0.2 × body length, flagellum 6–articulate, peduncle article 1 width less than 2 × article 2, accessory flagellum 2–articulate. Antenna 2 0.2 × body length, subequal in length with antenna 1, flagellum 4–articulate. Mandibular palp ratio of articles 1–3 1.0: 3.5: 1.6, article 2 robust with 4 setae, article 3 with 2 distal setae, incisors strongly dentate; left mandible with 12 raker spines, lacinia mobilis large, strongly toothed; right mandible with 12 raker spines, lacinia mobilis small, weakly dentate, with 3 rows of dentition. Upper lip asymmetrically lobate, anterior margin bare. Lower lip inner lobes fused, setose, with facial setae; outer lobes with moderate gape, anterior margins setose. Maxilla 1 palp 1–articulate with 3 distal setae; outer plate with 6 distal robust setae and 5 distal slender setae. Maxilla 2 inner plate with 3 robust distal setae and 5 slender distal setae; outer plate with 4 robust distal marginal setae and 21 marginal setae. Maxilliped inner plates distal margin with arc-shaped indentation, with short robust setae and long plumose setae; outer plate inner margin tuberculate, reaching 0.2 × palp article 1, inner plate plumose marginal setae; palp article 4 subequal in length with article 3, distally acute.

Pereon. Coxae 1–4 relative widths 1.0: 1.6: 1.4: 1.7. Gnathopod 1 coxa smooth, with tiny marginal setae, anterodistal margin produced, subquadrate with cusp, distal margin rounded, posteroventral margin narrowly rounded, facial setae absent; basis centrally expanded, anterior margin with 2 short setae, posterior margin with 2 medium setae; ischium bare; carpus linear, length 14.2 × width, proximal margin dentate, distal margin with 3 short setae; propodus curved, palm dentate with 6 distal setae; dactylus with linear striation and 1 short seta, reaching 0.2 × propodus length. Gnathopod 2 coxa broader than long, subequal to coxa 3, smooth, with tiny marginal setae, anterior margin expanded anteriorly with cusp, anterodistally subquadrate, distal margin straight, posterior margin straight, facial setae absent; basis distally expanded, anterior margin with 6 short setae, posterior margin bare; ischium with 3 distal setae and 1 posterodistal seta; carpus 0.3 × propodus length, curved, distally tapered, anterior margin dentate; propodus with 1 mediofacial setal row displaced to palm, reaching 0.7 × propodus length, with 1 row of robust submarginal setae, posterior margin smooth, palm convex with 4 major tubercles; dactylus curved, proximal margin smooth with 2 setae, distal margin with 1 tooth, anterior margin distally acute, reaching 0.7 × propodus length. Pereopod 3 coxa length 1.1 × width, anterodistal corner overriding distal face of coxa 2, extending below it, smooth, with tiny marginal setae, anterior margin expanded, distal margin slightly convex with cusps, posterior margin straight, facial setae absent. Pereopod 4 coxa smooth, with tiny marginal setae, anterior margin produced with cusp, distal margin evenly rounded, posterior margin excavate, facial setae absent. Pereopods 5–7 coxae facial setae absent; bases oval, width length ratios 1: 1.3, 1: 1.4, 1: 1.4; posterior margins serrate, setose.

Pleon. Epimera 1–2 with ventral setae, epimeron 3 bare; epimeron 3 posteroventral corner with small sinus, subquadrate. Uropods 1–3 relative lengths 1.0: 0.7: 0.7; inner and outer rami lined with short marginal setae. Uropod 1 peduncle and outer ramus subequal in length with inner ramus; inner and outer rami each with 3 robust setae. Uropod 2 peduncle 0.9 × inner ramus length; outer ramus 0.8 × inner ramus length; inner and outer rami each with 2 robust setae. Uropod 3 peduncle 1.5 × inner ramus length; outer ramus 0.9 × inner ramus length; inner and outer rami each with 2 robust setae. Telson 1.9 × longer than wide, without facial or marginal setae, apex very weakly tridentate.

##### Female (sexually dimorphic characters).

Gnathopod 1 basis anterior margin with 3 short setae, posterior margin with 15 medium setae; ischium with 1 posterodistal seta; carpus distal margin with 4 short setae; propodus palm with 9 distal setae. Gnathopod 2 propodus with longer robust submarginal setae.

**Figure 5:**
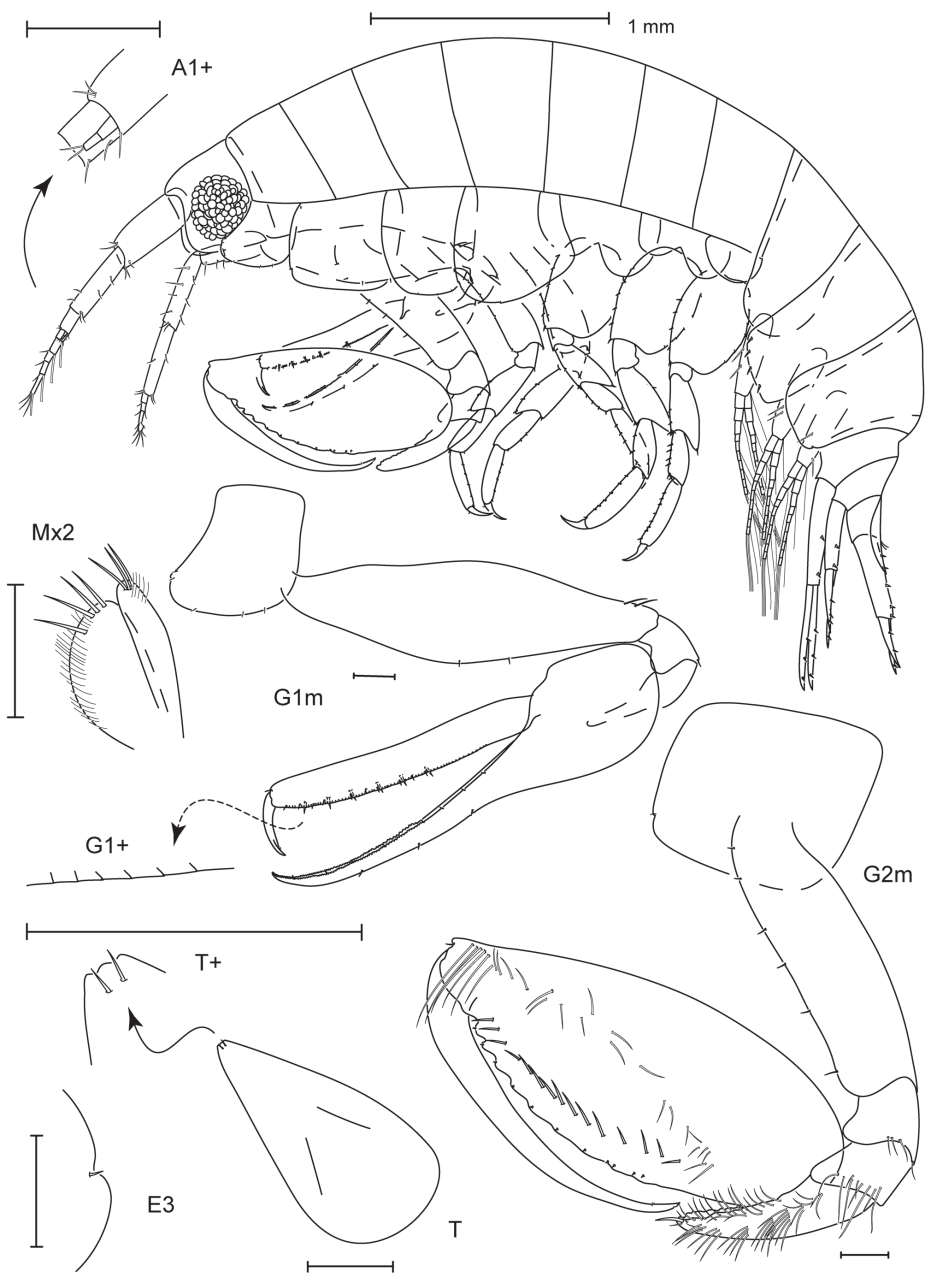
. *Leucothoe elegans* sp. n., holotype male, 4.5 mm, RUMF-ZC-1658.

**Figure 6.**
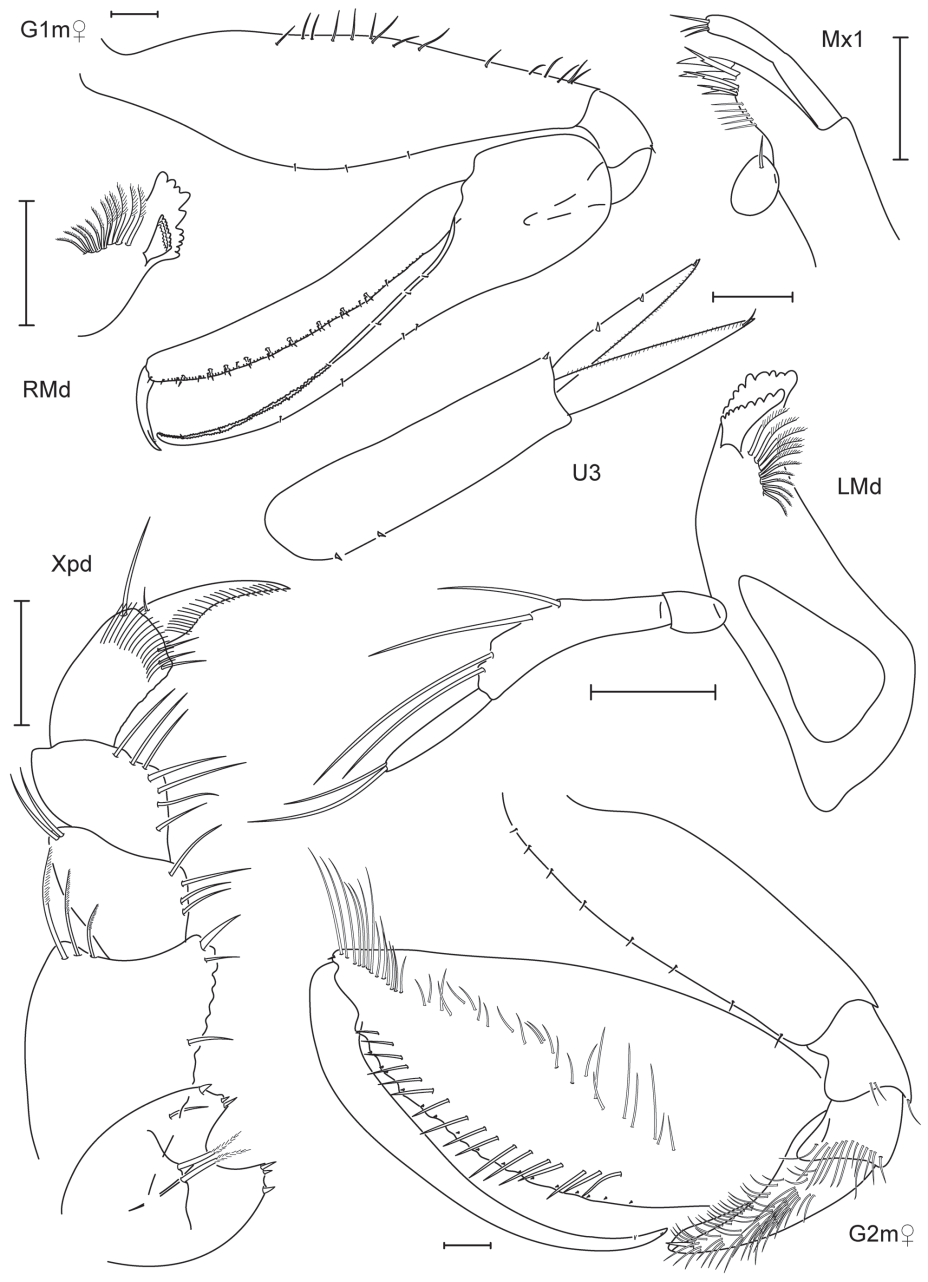
*Leucothoe elegans* sp. n., holotype male, 4.5 mm, RUMF-ZC-1658; paratype female, 5.7 mm, RUMF-ZC-1659.

##### Etymology.

After the Latin ‘elegans’, meaning tasteful, choice, fine, and referring to the elegant, elongate gnathopod 1 of males and females of this species.

##### Ecology.

In branchial chamber of solitary ascidian, *Rhopalaea circula* (Fig. 18F); grey/purple hard sponge; dark red chimney sponge; orange flame sponge; purple brown soft sponge; and orange stubby sponge.

##### Relationships.

*Leucothoe elegans* is similar to *Leucothoe germanalcyone* Hirayama, 1992 in having an enlarged eye; similar to *Leucothoe flammosa* Thomas and Klebba, 2007 and *Leucothoe uschakovi* Gurjanova, 1951 in having an elongate gnathopod 1 with a centrally widened basis; and similar to *Leucothoe nagatai* in having short antennae, narrow pereopod 5–7 bases, and a sinuous epimeron 3. *Leucothoe elegans* differs from these species in having a dentate right mandible lacinia mobilis, female gnathopod 1 with many posterior setae, and gnathopod 2 propodus with submarginal row of robust setae.

##### Remarks.

*Leucothoe elegans* is translucent ivory in color (Fig. 17C). This species has only been collected on Yakushima Island and from Shioya Bay, on the east coast of Okinawa–jima Island, Okinawa.

##### Distribution.

East China Sea: Okinawa–jima Island, Okinawa and Yakushima Island, Kagoshima, Japan.

#### Leucothoe nathani sp. n.

urn:lsid:zoobank.org:act:ECB34479-0D69-4027-8604-CE98C89F809B

http://species-id.net/wiki/Leucothoe_nathani

Figs 7 8

##### Type material.

Holotype male, 4.8 mm, RUMF-ZC-1663, Mizugama reef wall (26°21'35"N, 127°44'22"E), in branchial chamber of solitary ascidian, *Herdmania* of Lahille, 1888, 7–9 m, N.S. White col., 26 February 2011 (KNWOkinawa34J). Paratype female, 6.3 mm, RUMF-ZC-1664, same station data as holotype.

##### Type locality.

Mizugama, Okinawa, Japan (26°21'35"N, 127°44'22"E).

##### Additional Material Examined.

2 specimens, RUMF-ZC-1665, KNWOkinawa34J; 1 specimen, KNWOkinawa42G.

##### Diagnosis (male).

Maxilla 1 palp 1–articulate, margins constricted. Maxilliped outer plate reaching 0.4 × length of palp article 1. Male gnathopod 1 basis posterodistally expanded; carpus basally inflated; dactylus very short, reaching 0.1 × propodus length. Gnathopod 2 propodus mediofacial setal row very robust with tufts of setae, palm with 4 long tubercles and 1 large indentation.

##### Description (male).

Head. Anterior margin rounded, anterodistal margin evenly rounded; ventral cephalic keel anterior margin excavate, anteroventral margin subquadrate, ventral margin straight; eyes with more than 10 ommatidia, oval. Antenna 1 0.3 × body length, flagellum 6–articulate, peduncle article 1 width less than 2 × article 2, accessory flagellum absent, aesthetascs present. Antenna 2 0.3 × body length, subequal in length with antenna 1, flagellum 5–articulate. Mandibular palp ratio of articles 1–3, 1.0: 3.8: 1.8, article 2 with 6 setae, article 3 with 2 distal setae, incisors strongly dentate; left mandible with 10 raker spines, lacinia mobilis large, strongly toothed; right mandible with 9 raker spines, lacinia mobilis small, weakly dentate. Upper lip asymmetrically lobate, anterior margin setose. Lower lip inner lobes fused, bare; outer lobes with moderate gape, anterior margins setose. Maxilla 1 palp 1–articulate, margins constricted, with 4 distal setae; outer plate with 6 distal robust setae and 3 distal slender setae. Maxilla 2 inner plate with 3 short distal robust setae, 3 distal slender setae, and facial setae; outer plate 3 distal serrate robust setae, 7 marginal slender setae, and facial setae. Maxilliped inner plates distal margin with v-shaped indentation, with short robust setae and long setae, with facial setae; outer plate inner margin smooth, reaching 0.4 × length of palp article 1, with 6 distal setae and 1 distal robust seta, facial setae present; palp article 4 elongate, distally acute.

Pereon. Coxae 1–4 relative widths 1.0: 1.0: 0.8: 1.4. Gnathopod 1 coxa smooth, with tiny marginal setae, anterodistal corner produced, subtriangular, distal margin straight, posterior margin excavate, facial setae absent; basis posterodistally expanded, anterior margin with 5 short setae, posterior margin bare; ischium bare; carpus basally inflated, length 7.6 × width, proximal margin smooth, distal margin with 4 medium setae; propodus straight, palm smooth with 10 distal setae; dactylus smooth, reaching 0.1 × propodus length. Gnathopod 2 coxa equally as long as broad, subequal in length with coxa 3, smooth, with tiny marginal setae, anterodistally rounded, distal margin straight, posterior margin straight, facial setae absent; basis distally expanded, stout, with 3 small anterodistal tubercles, anterior margin with 9 short-medium length setae, posterior margin with 1 seta; ischium with 2 posterodistal setae; carpus 0.4 × propodus length, straight, distally tapered, anterior margin dentate; propodus with 1 mediofacial setal row displaced to palm, reaching 0.7 × propodus length, with 1 row of submarginal setae, posterior margin smooth, palm convex with 4 major tubercles; dactylus recurved, proximal margin smooth, bare, anterior margin distally acute, reaching 0.5 × propodus length. Pereopod 3 coxa length 1.4 × width, anterodistal corner overriding distal face of coxa 2, not extending below it, smooth, bare, anterior margin straight, distal margin oblique, posterior margin straight, facial setae absent. Pereopod 4 coxa smooth, bare, anterior margin produced, distal margin evenly rounded, posterior margin tapered, facial setae absent. Pereopod 5 coxa facial seta present, pereopods 6–7 coxae facial setae absent; bases oval, width length ratios 1:1.6, 1:1.6, 1:1.5, posterior margins smooth, setose.

Pleon. Epimera 1–2 with ventral setae, epimeron 3 bare; epimeron 3 posteroventral corner rounded. Uropods 1–3 relative lengths 1.0: 0.6: 0.8; inner and outer rami lined with short marginal setae. Uropod 1 peduncle 1.1 × inner ramus length; outer ramus subequal in length with inner ramus; inner ramus with 6 robust seate; outer ramus with 3 robust setae. Uropod 2 peduncle 0.7 × inner ramus length; outer ramus 0.8 × inner ramus length; inner ramus with 1 robust seta; outer ramus with 3 robust seate. Uropod 3 peduncle 1.4 × inner ramus length; outer ramus subequal in length with inner ramus; inner and outer rami each with 1 robust seta. Telson 2.2 × longer than wide, apex weakly tridentate.

##### Female (sexually dimorphic characters).

Gnathopod 1 basis anterior margin with 15 short setae; carpus linear, distal margin with 7 longer setae. Gnathopod 2 basis anterior margin with 21 short setae; carpus anterior margin smooth; dactylus with two proximal setae.

**Figure 7.**
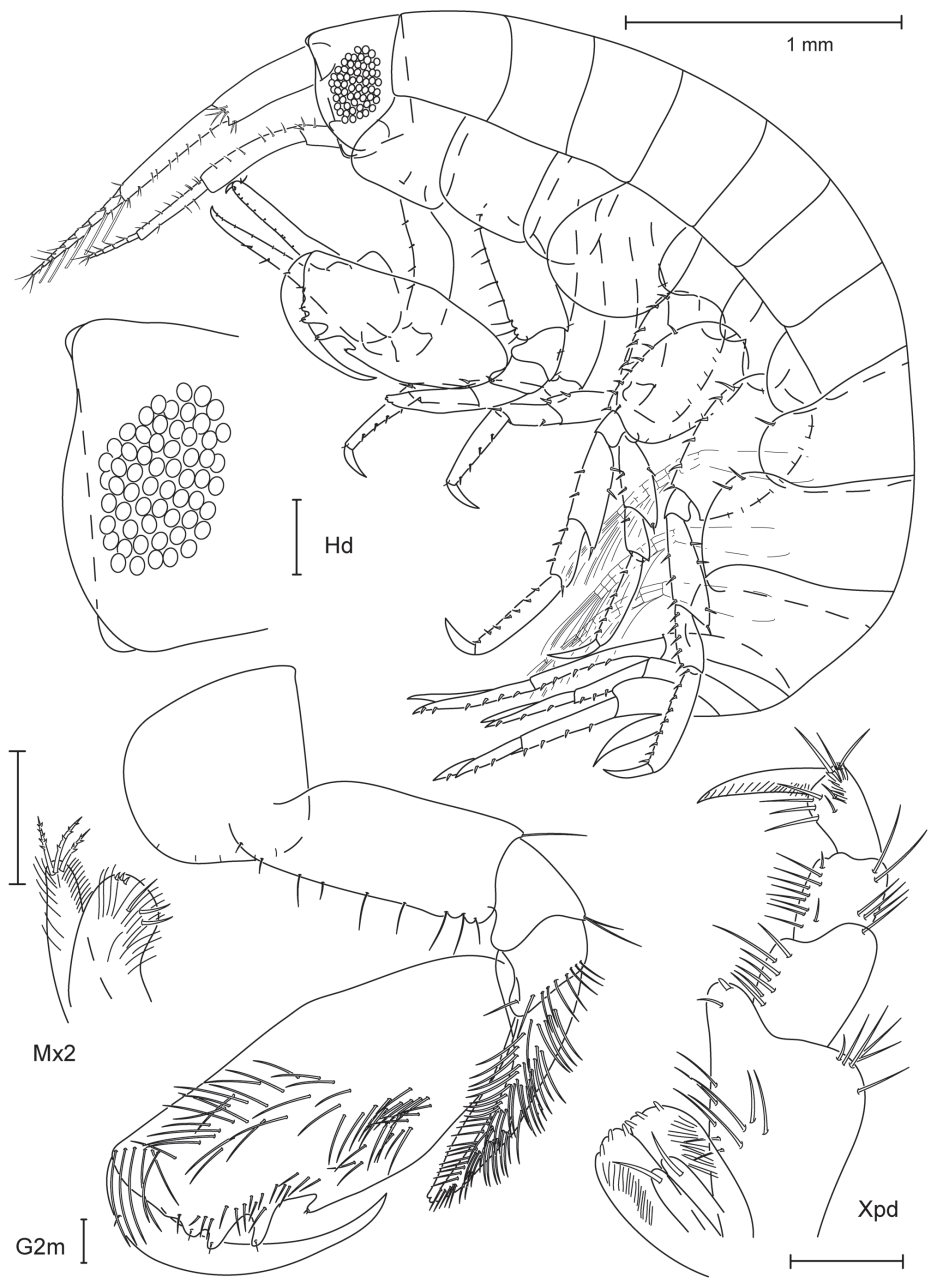
*Leucothoe nathani* sp. n., holotype male, 4.8 mm, RUMF-ZC-1663.

**Figure 8.**
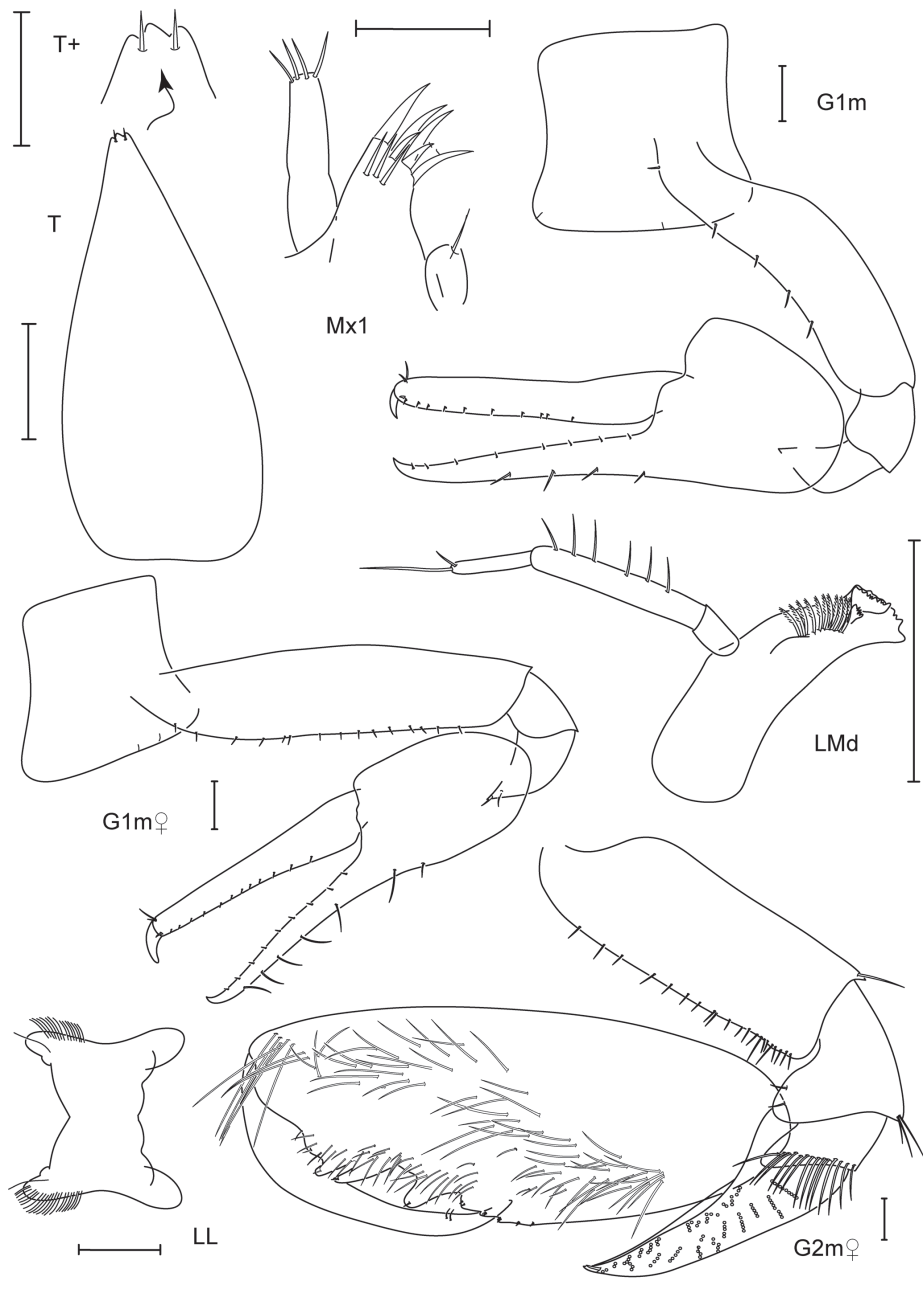
*Leucothoe nathani* sp. n., holotype male, 4.8 mm, RUMF-ZC-1663; paratype female, 6.3 mm, RUMF-ZC-1664.

##### Etymology.

Named for Nathan Stuart White, amphipod collector extraordinaire, who collected the type specimens of this species. Nathan has provided tremendous support and assistance throughout all sampling efforts in the Ryukyu Archipelago.

##### Ecology.

In branchial chamber of solitary ascidian, *Herdmania* (Fig. 18A) and compound ascidian, *Clavelina* of Savigny, 1816 (Fig. 18C).

##### Relationships.

*Leucothoe nathani* is similar to *Leucothoe nagatai* in having short antennae, an elongate maxilliped outer plate inner margin, a short gnathopod 1 dactyl, a heavily setose gnathopod 2 propodus medial surface, and narrow pereopod 5-7 bases. This species differs in having a smooth maxilliped outer plate inner margin, slenderner gnathopod 1 carpus (length 7.6 × width compared to length 6.6 × width in *Leucothoe nagatai*), and a longer telson (2.2 × longer than wide compared to 1.8 × longer than wide in *Leucothoe nagatai*).

##### Remarks.

*Leucothoe nathani* is orange in color with robust dark orange stripes along pereonites (Fig. 17D). This species has been collected from only one location in February and April, two of the coldest months on Okinawa–jima Island.

##### Distribution.

East China Sea: Okinawa–jima Island, Japan.

#### Leucothoe obuchii sp. n.

urn:lsid:zoobank.org:act:590FEF5F-1261-4F6E-B4F5-07B7940CD81B

http://species-id.net/wiki/Leucothoe_obuchii

Figs 9 10

##### Type material.

Holotype male, 4 mm, RUMF-ZC-1666, Tettou–mae–oki, Oura–wan Bay (26°32'43"N, 128°02'56"E), muddy sand slope, in branchial chamber of solitary ascidian *Rhopalaea* of Phillippi, 1843 (clear with black and yellow lines), 24 m, M. Obuchi, col., 4 March 2011 (KNWOkinawa37A). Paratype female, 3.8 mm, RUMF-ZC-1667, same station data as holotype.

##### Type locality.

Tettou–mae–oki, Oura–wan Bay, Okinawa, Japan (26°32'43"N, 128°02'56"E).

##### additional material examined.

5 specimens, RUMF-ZC-1668, KNWAmami3F; 2 specimens, NSMT-Cr21817, KNWOkinawa48A; 2 specimens, NSMT –Cr21818, KNWYaku3K; 1 specimen, NSMT –Cr21819, KNWYaku5N.

##### Diagnosis (male).

Maxilla 1 palp 1–articulate. Maxilliped outer plate inner margin tuberculate, reaching 0.7 × palp article 1. Gnathopod 1 basis centrally widened; carpus with 4 long distal setae; propodus inflated; dactylus short, reaching 0.2 × propodus length. Pereopods 5–7 bases narrow, oval; epimeron 3 posteroventral margin with small sinus.

##### Description (male).

Head. Anterior margin rounded, anterodistal margin evenly rounded; ventral cephalic keel anterior margin transverse, anteroventral margin rounded, ventral margin excavate; eyes with more than 10 ommatidia, oval. Antenna 1 0.3 × body length, flagellum 7–articulate, peduncle article 1 width less than 2 × article 2, accessory flagellum 1–articulate, aesthetascs present. Antenna 2 0.2 × body length, shorter than antenna 1, flagellum 3–articulate. Mandibular palp ratio of articles 1–3 1.0: 2.5: 1.2, article 2 and 3 each with 2 distal setae, incisors strongly dentate; left mandible with 7 raker spines, lacinia mobilis large, strongly toothed; right mandible with 8 raker spines, lacinia mobilis small, weakly dentate. Upper lip asymmetrically lobate, anterior margin setose. Lower lip inner lobes fused, setose; outer lobes with moderate gape, anterior margins setose, with facial setae. Maxilla 1 palp 1–articulate with 3 distal setae; outer plate with 6 distal robust setae and 3 distal slender setae. Maxilla 2 inner plate with 4 distal and 4 marginal setae; outer plate with 5 robust distal setae and 4 marginal setae. Maxilliped inner plates distal margin with v-shaped indentation, with short robust setae; outer plate inner margin tuberculate, reaching 0.7 × palp article 1, with 1 simple distal seta; palp article 4 subequal in length with article 3, distally acute.

Pereon. Coxae 1–4 relative widths 1.0: 1.5: 1.2: 1.8. Gnathopod 1 coxa smooth, bare, anterodistal corner produced, subquadrate with cusp, distal margin straight, posterior margin excavate, facial setae absent; basis centrally widened, anterior margin with 2 short setae, posterior margin with 2 short setae; ischium bare; carpus linear, length 7.1 × width, proximal margin dentate, distal margin with 4 long setae; propodus curved, slightly inflated, palm smooth with 3–5 distal setae; dactylus smooth, reaching 0.2 × propodus length. Gnathopod 2 coxa broader than long, subequal to coxa 3, smooth, bare, anterodistally rounded, distal margin straight, posterior margin straight, facial setae absent; basis distally expanded, anterior margin with 4 setae, posterior margin bare; ischium bare; carpus 0.4 × propodus length, straight, distally tapered, anterior margin with indentation; propodus with 1 mediofacial setal row displaced below midline, reaching 0.7 × propodus length, with 1 row of submarginal setae, posterior margin smooth, palm convex with 4 major tubercles; dactylus curved, proximal margin smooth, bare, anterior margin distally acute, reaching 0.4 × propodus length. Pereopod 3 coxa length 1.2 × width, anterodistal corner overriding distal face of coxa 2, extending below it, smooth, bare, anterior margin expanded, distal margin straight, posterior margin straight, facial setae absent. Pereopod 4 coxa smooth, bare, anterior margin tapered with cusp, distal margin evenly rounded, posterior margin tapered, facial setae absent. Pereopods 5–7 coxae facial setae absent; bases oval, width length ratios 1: 1.5, 1: 1.5, 1:1.5, posterior margins smooth, setose.

Pleon. Epimeron 1 bare; epimera 2–3 with ventral setae; epimeron 3 posteroventral corner slightly sinuous, rounded. Uropods 1–3 relative lengths 1.0: 0.7: 0.7; inner and outer rami lined with short marginal setae. Uropod 1 peduncle and outer ramus subequal in length with inner ramus; inner ramus with 1 robust seta; outer ramus with 3 robust setae. Uropod 2 peduncle subequal in length with inner ramus; outer ramus 0.8 × inner ramus length; inner ramus with 1 robust seta; outer ramus with 2 robust setae. Uropod 3 peduncle 1.4 × inner ramus length; outer ramus 0.8 × inner ramus length; inner and outer rami without robust setae. Telson 1.9 × longer than wide, apex weakly tridentate.

##### Female (sexually dimorphic characters).

Gnathopod 1 basis anterior margin with 4 setae, posterior margin with 1 posterodistal seta. Gnathopod 2 basis anterior margin with 1 seta; carpus anterior margin smooth.

**Figure 9.**
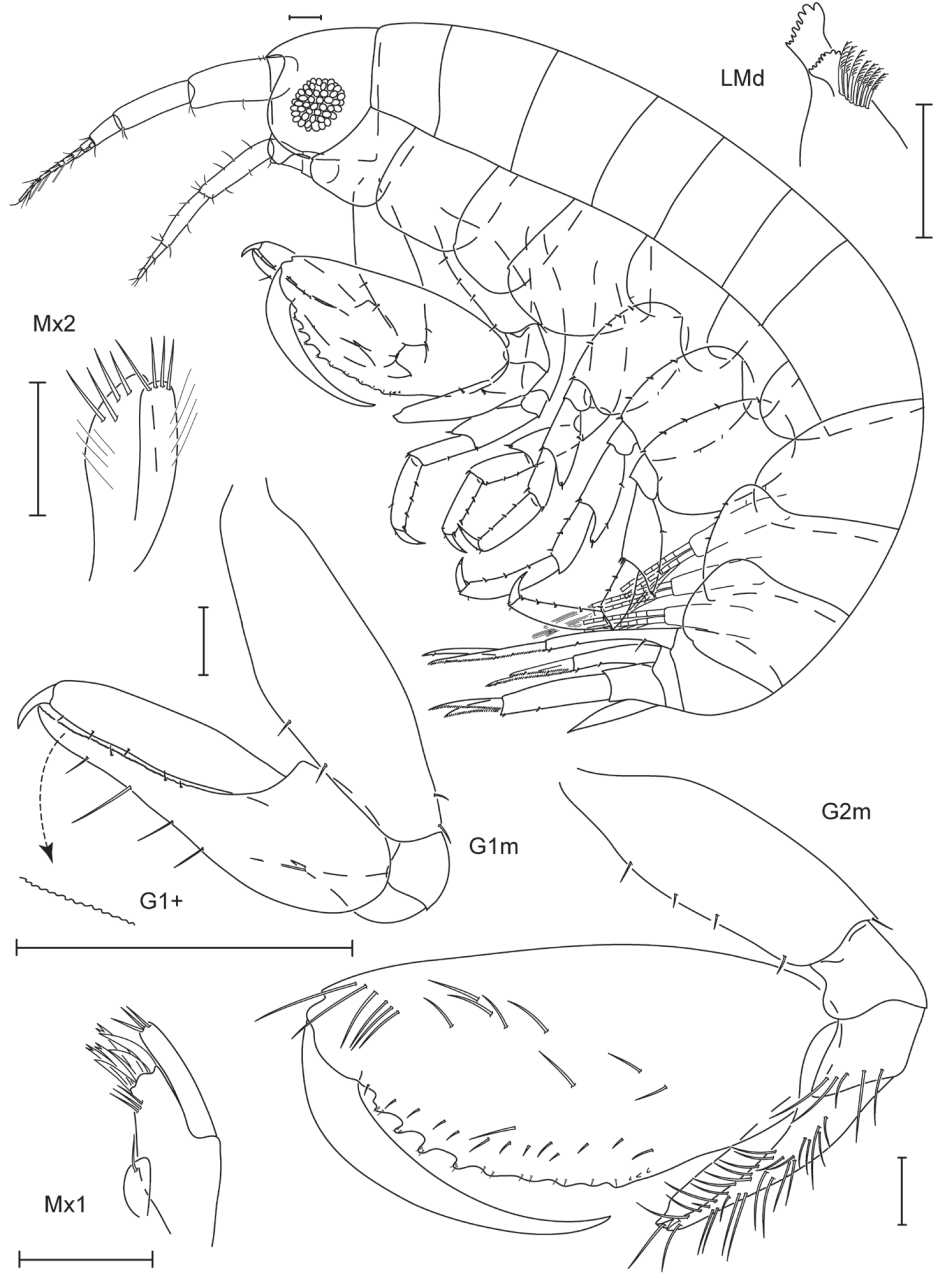
*Leucothoe obuchii* sp. n., holotype male, 4.0 mm, RUMF-ZC-1666.

**Figure 10.**
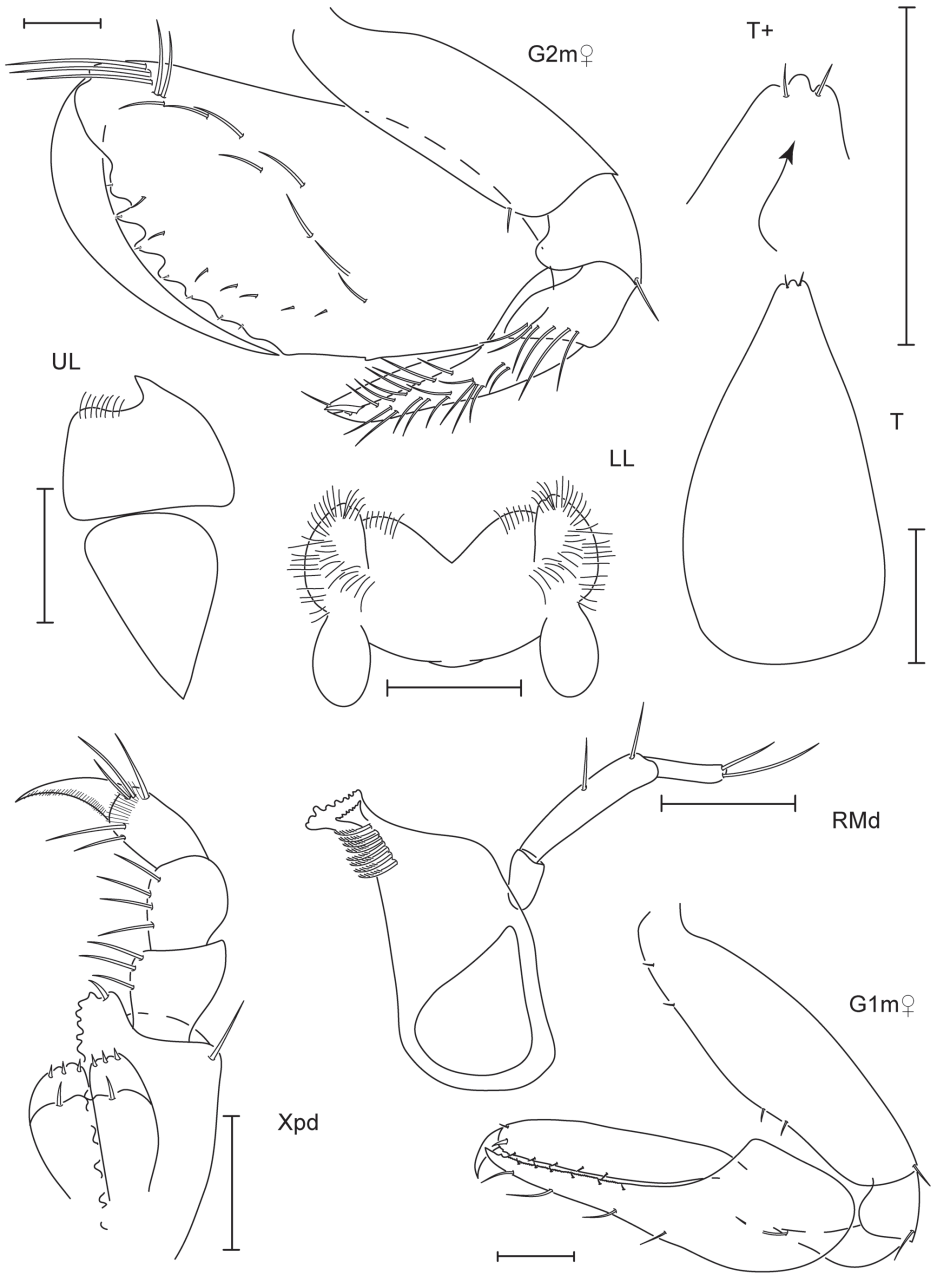
*Leucothoe obuchii* sp. n., holotype male, 4.0 mm, RUMF-ZC-1666; paratype female, 3.8 mm, RUMF-ZC-1667.

##### Etymology.

Named for “General” Masami Obuchi, who collected the type specimens of this species. Dr. Obuchi has provided invaluable sampling and logistical support for this research in the Ryukyu Archipelago.

##### Ecology.

In branchial chamber of solitary ascidians, *Rhopalaea* (Fig. 18B) and *Rhopalaea circula* (Fig. 18F); and coral rubble.

##### Relationships.

*Leucothoe obuchii* is similar to *Leucothoe nagatai* in having short antennae, an elongate tuberculate maxilliped outer plate inner margin, a short gnathopod 1 dactyl, and narrow pereopod 5-7 bases. This species differs in having a much less setose gnathopod 2 propodus medial surface. *Leucothoe nagatai* has robust tufts of mediofacial setae covering most of the proximal surface of the propodus compared to single mediofacial and submarginal setal rows in *Leucothoe obuchii*.

##### Remarks.

*Leucothoe obuchii* is opaque ivory in color (Fig. 17E). In most collections of this species there was one specimen at the base of the branchial chamber of each ascidian collected. Rarely, there were one large and one small amphipod living together.

##### Distribution.

East China Sea: Okinawa–jima Island (Okinawa), Tokunoshima Island, Amami–oshima Island, and Yakushima Island (all Kagoshima), Japan.

#### Leucothoe trulla sp. n.

urn:lsid:zoobank.org:act:211A291A-EBDE-47F1-86F6-3BE9D376049D

http://species-id.net/wiki/Leucothoe_trulla

Figs 11 12

##### Type material.

Holotype male, 4.3 mm, RUMF-ZC-1669, Inoda Beach patch reef (24°27'46"N, 124°15'13"E), coral rubble, K.N. White col. 20 April 2011 (KNWIshigaki3G). Paratype female, 4.4 mm, RUMF-ZC-1670, same station data as holotype.

##### Type locality.

Inoda Beach, Ishigaki, Japan (24°27'46"N, 124°15'13"E).

##### Additional material examined.

2 specimens, RUMF-ZC-1671, KNWIriomote4A; 3 specimens, NSMT –Cr21820, KNWIshigaki3G.

##### Diagnosis (male).

Maxilla 1 palp 1–articulate, margins constricted. Gnathopod 1 coxa posterior margin distally serrate. Gnathopod 2 coxa distal margin serrate; carpus distally truncate, spoon-like; propodus mediofacial setal row displaced to midline.

##### Description (male).

Head. Anterior margin rounded, anterodistal margin evenly rounded; ventral cephalic keel anterior margin excavate, anteroventral margin rounded with an anteriorly projecting cusp, ventral margin straight; eyes with more than 10 ommatidia, round. Antenna 1 0.4 × body length, flagellum 10–articulate, peduncle article 1 width less than 2 × article 2, accessory flagellum 1–articulate, aesthetascs present. Antenna 2 0.3 × body length, shorter than antenna 1, flagellum 5–articulate. Mandibular palp ratio of articles 1–3, 1.0: 2.7: 1.4, article 2 with 5–6 setae, article 3 with 2 distal setae, incisors weakly dentate; left mandible with 7 raker spines, lacinia mobilis large, strongly toothed; right mandible with 7 raker spines, lacinia mobilis small, weakly dentate. Upper lip asymmetrically lobate, anterior margin setose. Lower lip inner lobes fused, bare; outer lobes with moderate gape, anterior margins setose. Maxilla 1 palp 1–articulate, margins constricted, with 3 distal setae; outer plate with 7 distal robust setae. Maxilla 2 inner plate with 5 distal setae, 3 marginal setae, and facial setae; outer plate with 4 robust distal setae and 15 marginal setae. Maxilliped inner plates distal margin with v-shaped indentation, with short robust setae and short setae; outer plate inner margin smooth, reaching 0.2 × length of palp article 1, with simple and plumose marginal setae, facial setae absent; palp article 4 subequal in length with article 3, distally acute.

Pereon. Coxae 1–4 relative widths 1.0: 1.3: 1.1: 1.8. Gnathopod 1 coxa smooth, with tiny marginal setae, smooth, anterodistal margin produced, subquadrate with cusp, distal margin straight, posterior margin excavate, distally serrate, facial setae absent; basis linear, anterior margin with 6 setae, posterior margin bare; ischium bare; carpus linear, length 19.8 × width, proximal margin dentate, distal margin with 3 short setae; propodus straight, palm dentate with 5 distal setae; dactylus smooth, reaching 0.4 × propodus length. Gnathopod 2 coxa longer than broad, subequal to coxa 3, smooth, with tiny marginal setae, distal margin serrate, anterodistally rounded, distal margin straight, posterior margin straight, facial setae absent; basis distally expanded, anterior margin with 6 short setae, posterior margin bare; ischium bare; carpus 0.3 × propodus length, curved, distally truncate, spoon-like, anterior margin smooth; propodus with 1 slightly displaced mediofacial setal row, reaching 0.6 × propodus length, with 1 row of submarginal setae, posterior margin smooth, palm convex with 3 major and 2 minor tubercles; dactylus curved, proximal margin smooth, bare, anterior margin distally subacute, reaching 0.7 × propodus length. Pereopod 3 coxa length 1.3 × width, anterodistal corner overriding distal face of coxa 2, extending below it, smooth, bare, anterior margin straight, distal margin oblique, posterior margin straight, facial setae absent. Pereopod 4 coxa smooth, bare, anterior margin straight, distal margin evenly rounded, posterior margin excavate, facial setae absent. Pereopods 5–7 coxae facial setae absent; bases slightly posteriorly tapered, width length ratios 1: 1.3, 1: 1.3, 1: 1.4; posterior margins smooth, bare.

Pleon. Epimera 1–3 with ventral setae; epimeron 3 posteroventral corner rounded. Uropods 1–2 relative lengths 1.0: 0.8. Uropod 1 peduncle 0.9 × inner ramus length; outer ramus 0.9 × inner ramus length; inner ramus with 5 robust seta; outer ramus with 8 robust setae. Uropod 2 peduncle 0.9 × inner ramus length; outer ramus 0.7 × inner ramus length; inner and outer rami each with 4 robust setae. Uropod 3 missing. Telson 2.3 × longer than wide, apex weakly tridentate.

##### Female (sexually dimorphic characters).

Gnathopod 1 basis anterior margin with 5 setae, posterior margin with 4 setae; carpus distal margin with 2 short setae. Gnathopod 2 basis anterior margin with 13 short and medium setae; ischium with 2 distal setae; carpus truncate, not spoon-like; propodus palm with smaller tubercles. Uropod 3 peduncle 0.7 × inner ramus length; outer ramus broken; inner and outer rami with robust setae.

**Figure 11.**
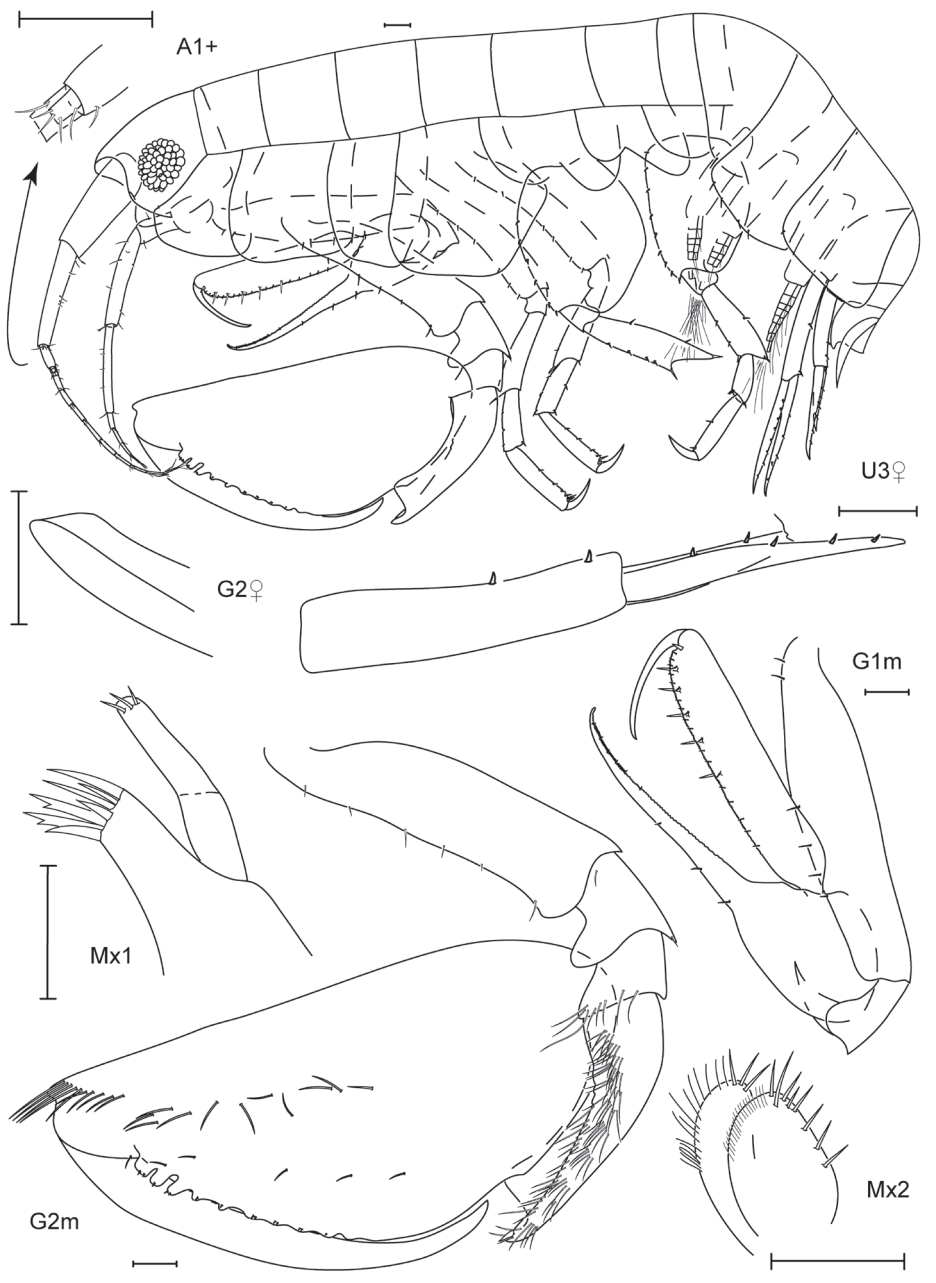
*Leucothoe trulla* sp. n., holotype male, 4.3 mm, RUMF-ZC-1669; paratype female, 4.4 mm, RUMF-ZC-1670.

**Figure 12.**
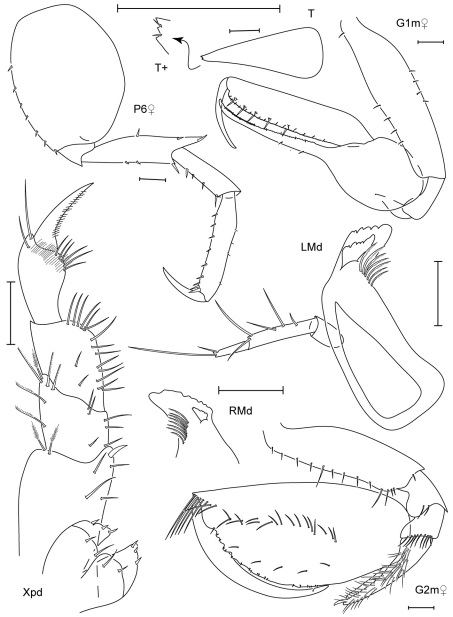
*Leucothoe trulla* sp. n., holotype male, 4.3 mm, RUMF-ZC-1669; paratype female, 4.4 mm, RUMF-ZC-1670.

##### Etymology.

After the Latin ‘trulla’, meaning ‘stirring spoon, skimmer’ and referring to the spoon-like carpus on male gnathopod 2.

##### Ecology.

In branchial chamber of solitary ascidian, *Herdmania* (Fig. 18A); and coral rubble.

##### Relationships.

*Leucothoe trulla* is similar to *Leucothoe commensalis*, *Leucothoe wuriti*, *Leucothoe epidemos*, and *Leucothoe thula*. The members of this “*Leucothoe commensalis* group” share a rounded head, long gnathopod 1 dactylus, a displaced gnathopod 2 propodus mediofacial setal row, and wide pereopod 5-7 bases. This species is also similar to *Leucothoe alata*, *Leucothoe dentata* Ledoyer, 1973, *Leucothoe denticulata* Costa, 1853, *Leucothoe epidemos*, *Leucothoe lihue* Barnard, 1970, *Leucothoe rudicula* White and Thomas, 2009,and *Leucothoe tolkieni* Vinogradov, 1990 in having a spoon-like gnathopod 2 carpus. This species differs from all of these species in having a ventral cephalic keel with an excavate anterior margin; constricted margins on the maxilla 1 palp, a serrate coxa 1 posteroventral margin; a serrate distal coxa 2 margin; and a rounded epimeron 3 posteroventral corner.

##### Remarks.

*Leucothoe trulla* has faint pink-red stripes along pereonite edges and a slightly darker “saddleback” color in the middle (Fig. 17F). This species is endemic to the southern Ryukyu Islands.

##### Distribution.

East China Sea: Ishigaki–jima Island and Iriomote–jima Island, Okinawa, Japan.

#### Leucothoe vulgaris sp. n.

urn:lsid:zoobank.org:act:C8E57473-2175-403A-93E4-77F590BF5EBC

http://species-id.net/wiki/Leucothoe_vulgaris

Figs 13 14

##### Type material.

Holotype male, 4.8 mm, RUMF-ZC-1672, Zanpa Cape reef wall (26°26'27.19"N, 127°43'03"E), in branchial chamber of solitary ascidian, *Pyura* of Molina, 1782, 10–30 m, K.N. White and N.S. White col., 13 December 2010 (KNWOkinawa23A). Paratype female, 4.2 mm, RUMF-ZC-1673, same station data as holotype.

##### Type locality.

Zanpa Cape, Okinawa, Japan (26°26'27"N, 127°43'03"E).

##### Additional Material Examined.

1 specimen, RUMF-ZC-1674, KNWOkinawa12F ; 1 specimen, RUMF-ZC-1675, KNWOkinawa11E ; 1 specimen, RUMF-ZC-1676, KNWJap10-9-8A ; 1 specimen, RUMF-ZC-1677, KNWOkinawa14H; 1 specimen, RUMF-ZC-1678, KNWOkinawa16E; 1 specimen, RUMF-ZC-1679, KNWOkinawa21F; 2 specimens, RUMF-ZC-1680, KNWOkinawa26A; 2 specimens, RUMF-ZC-1681, KNWOkinawa24B; 1 specimen, RUMF-ZC-1682, KNWOkinawa25F; 3 specimens, RUMF-ZC-1683, KNWOkinawa27C; 2 specimens, RUMF-ZC-1684, KNWOkinawa27B; 1 specimen, RUMF-ZC-1685, KNWOkinawa29A; 2 specimens, RUMF-ZC-1686, KNWOkinawa29E; 1 specimen, RUMF-ZC-1687, KNWOkinawa31D; 1 specimen, RUMF-ZC-1688, KNWOkinawa36B; 1 specimen, RUMF-ZC-1689, KNWOkinawa36F ; 1 specimen, NSMT-Cr21821, KNWOkinawa37E; 2 specimens, NSMT –Cr21822, KNWOkinawa38A; 1 specimen, NSMT –Cr21823, KNWOkinawa39M; 12 specimens, NSMT –Cr21824, KNWOkinawa42F ; 1 specimen, NSMT - Cr21825, KNWIshigaki4E; 3 specimens, NSMT - Cr21826, KNWIriomote2A; 3 specimens, NSMT - Cr21827, KNWIriomote2D; 2 specimens, NSMT - Cr21828, KNWYaku3P; 1 specimen, NSMT - Cr21829, KNWYaku3Q; 2 specimens, NSMT - Cr21830, KNWOkinawa51B; 2 specimens, NSMT - Cr21831, KNWIshigaki2E; 8 specimens, NSMT - Cr21832, KNWIriomote2I ; 8 specimens, NSMT - Cr21833, KNWIriomote3D ; 1 specimen, NSMT - Cr21834, KNWYaku1L.

##### Diagnosis (male).

Mandibular palp article 2 with 15 setae. Right mandible lacinia mobilis with dentate surface. Upper lip epistome with marginal setae. Gnathopod 1 coxa with 1 long medial seta. Gnathopod 2 carpus distally truncate, expanded; propodus mediofacial setal row displaced below midline. Pereopods 5–7 bases posteriorly tapered. Epimeron 1 with anteroventral tuft of setae. Telson apex with strong point.

##### Description (male).

Head. Anterior margin rounded, anterodistal margin evenly rounded; ventral cephalic keel anterior margin excavate, anteroventral margin subquadrate, ventral margin straight; eyes with more than 10 ommatidia, round. Antenna 1 0.3 × body length, flagellum 9–articulate, peduncle article 1 width less than 2 × article 2, accessory flagellum absent, aesthetascs absent. Antenna 2 0.3 × body length, subequal in length with antenna 1, flagellum 3–articulate. Mandibular palp ratio of articles 1–3, 1.0: 3.4: 1.5, article 2 with 15 setae, article 3 with 2 distal setae, incisors strongly dentate; left mandible with 12 raker spines, lacinia mobilis large, strongly toothed; right mandible with 11 raker spines, lacinia mobilis small, with dentate surface. Upper lip asymmetrically lobate, anterior margin setose; epistome with marginal setae. Lower lip inner lobes fused, with facial setae; outer lobes with moderate gape, anterior margins setose. Maxilla 1 palp 2–articulate with 4 distal setae; outer plate with 7 distal robust setae and 4 distal slender setae. Maxilla 2 inner plate with 7 robust distal setae and 10 slender distal setae; outer plate with 3 robust distal setae and 13 slender marginal setae. Maxilliped inner plates distal margin with v-shaped indentation, with short robust setae and long setae; outer plate inner margin smooth, reaching 0.2 × length of palp article 1, with 4 distal setae and 1 distal spine, facial setae absent; palp article 4 subequal in length with article 3, distally acute.

Pereon. Coxae 1–4 relative widths 1.0: 1.1: 0.8: 1.5. Gnathopod 1 coxa smooth, with tiny marginal setae, smooth, anterodistal margin produced, subquadrate, serrate, distal margin straight, posterior margin excavate, medial facial seta present; basis proximally widened, anterior margin with 6 short setae, posterior margin bare; ischium bare; carpus linear, length 14.2 × width, proximal margin dentate, distal margin with 3 short setae; propodus straight, palm dentate with 6 distal setae; dactylus smooth, reaching 0.3 × propodus length. Gnathopod 2 coxa broader than long, subequal to coxa 3, smooth, with tiny marginal setae, anterodistally rounded, distal margin straight, posterior margin straight, facial setae absent; basis linear, with two small anterodistal tubercles, anterior margin with 11 short and long setae, posterior margin bare; ischium with 2 anterior setae; carpus 0.3 × propodus length, curved, distally truncate, expanded, anterior margin dentate; propodus with 1 mediofacial setal row displaced below midline, reaching 0.8 × propodus length, with 1 row of submarginal setae, posterior margin smooth, palm convex with 3 major tubercles; dactylus curved, proximal margin smooth, bare, anterior margin distally subacute, reaching 0.6 × propodus length. Pereopod 3 coxa length 1.5 × width, anterodistal corner overriding distal face of coxa 2, extending below it, smooth, with tiny marginal setae, anterior margin straight, distal margin oblique, posterior margin straight, facial setae absent. Pereopod 4 coxa smooth, bare, anterior margin produced, distal margin evenly rounded, posterior margin excavate, facial setae absent. Pereopods 5–7 coxae facial setae absent; bases posteriorly tapered, width length ratios 1: 1.3, 1: 1.3, 1: 1.1; posterior margins smooth, setose.

Pleon. Epimeron 1 with tuft of anteroventral setae, epimeron 2 with ventral setae, epimeron 3 bare; epimeron 3 posteroventral corner subquadrate. Uropods 1–3 relative lengths 1.0: 0.8: 1.1. Uropod 1 peduncle and outer ramus subequal in length with inner ramus; inner ramus with 4 robust seta; outer ramus with 8 robust setae. Uropod 2 peduncle 0.7 × inner ramus length; outer ramus 0.6 × inner ramus length; inner ramus with 3 robust seta; outer ramus with 2 robust setae. Uropod 3 peduncle 1.1 × inner ramus length; outer ramus 0.9 × inner ramus length; inner ramus with 2 robust seta; outer ramus with 5 robust setae. Telson 2.5 × longer than wide, apex with strong point.

##### Female (sexually dimorphic characters).

Gnathopod 1 basis anterior margin with 10 short setae; carpus distal margin with 4 short setae; propodus palm with 4 distal setae. Gnathopod 2 basis without tubercles, anterior margin with 13 short and long setae; ischium proximal ridge of 3 setae, 1 distal seta, and 1 posterodistal seta; carpus distal end slightly expanded; propodus palm with smaller tubercles.

**Figure 13.**
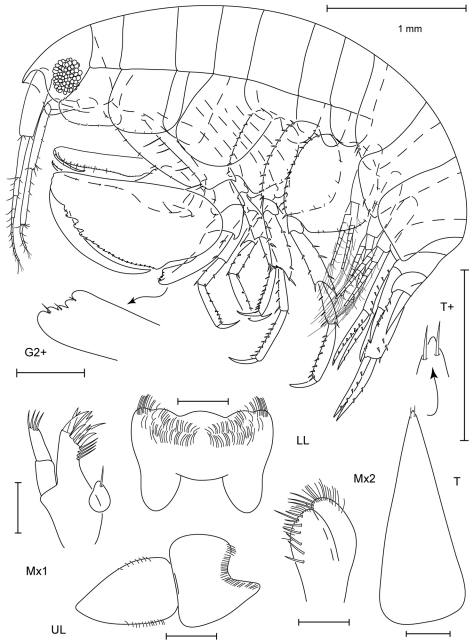
*Leucothoe vulgaris* sp. n., holotype male, 4.8 mm, RUMF-ZC-1672.

**Figure 14.**
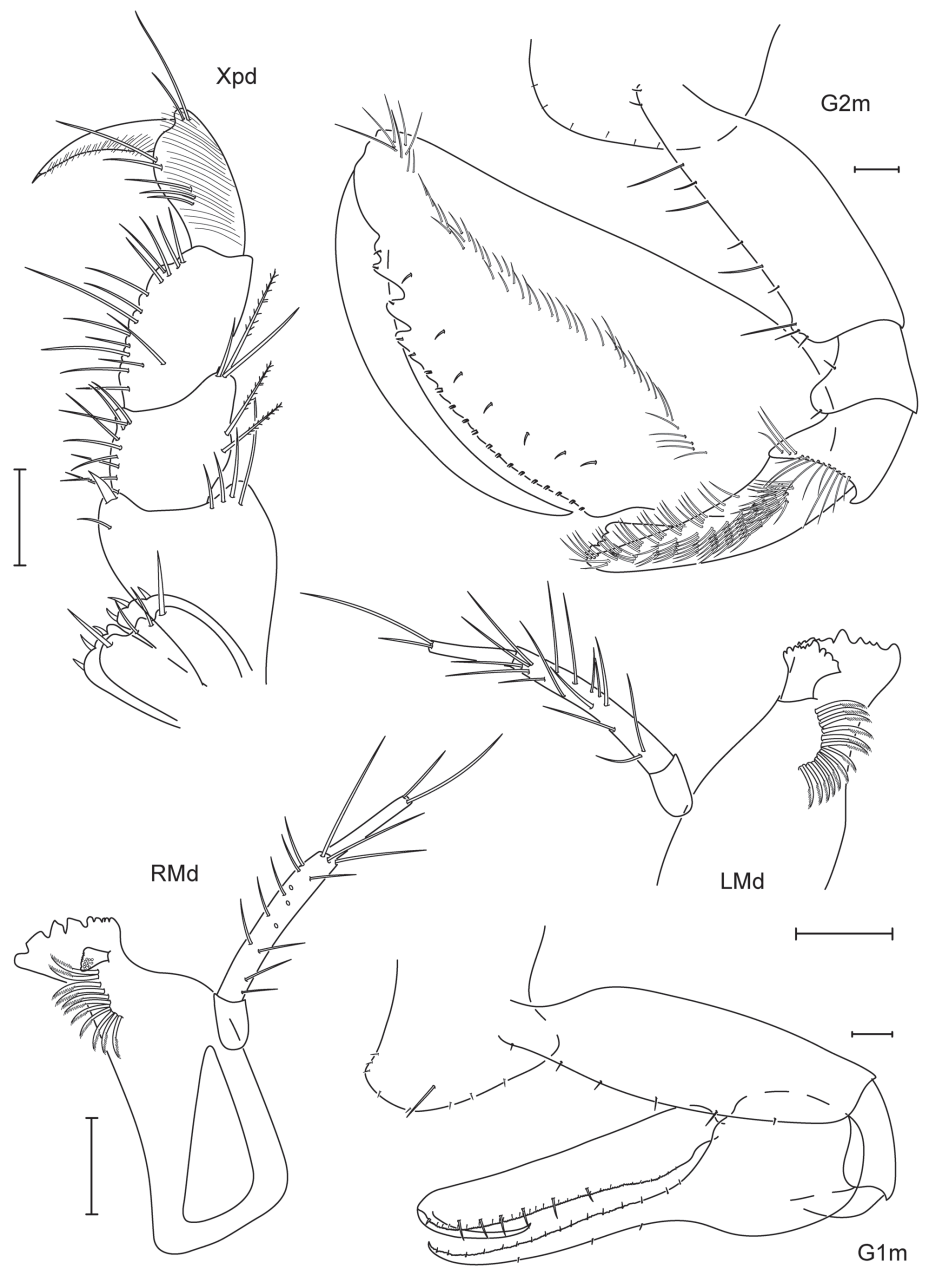
*Leucothoe vulgaris* sp. n., holotype male, 4.8 mm, RUMF-ZC-1673.

##### Etymology.

After the Latin ‘vulgaris’, meaning ‘common, commonplace’ and referring to the widespread distribution and the apparent lack of host specificity of this species.

##### Ecology.

In branchial chamber of solitary ascidians, *Pyura* sp. (Fig. 18E); *Pyura microcosmus* (Fig. 18D); *Rhopalaea circula* (Fig. 18F); compound ascidians, *Clavelina* sp. (Fig. 18C); purple hard sponge with small holes; *Haliclona* of Grant, 1836 (blue sponge); *Callyspongia* of Duchassaing & Michelotti, 1864 (beige sponge); and coral rubble.

##### Relationships.

*Leucothoe vulgaris* is part of the “*Leucothoe commensalis* group” in the same aspects that *Leucothoe trulla* is similar (see ‘Relationships’ under *Leucothoe trulla*). *Leucothoe vulgaris* differs from these species in having a setose epistome (smooth in reports of all *Leucothoe* species), a dentate surface on right mandible lacinia mobilis, and a telson with a strong point (most *Leucothoe* species have a tridentate apex).

##### Remarks.

*Leucothoe vulgaris* has a distinct red “saddleback” color pattern found in ascidian-dwelling leucothoids worldwide and yellow antennae (Fig. 17A). This species is widespread throughout the Ryukyu Archipelago, inhabiting many species of ascidians and sponges.

##### Distribution.

East China Sea: Okinawa–jima Island, Iriomote–jima Island, Ishigaki–jima Island (all Okinawa), Okinoerabu–jima Island, Yoron–jima Island, Tokunoshima Island, Amami–oshima Island, and Yakushima Island (all Kagoshima), Japan.

### Paranamixis

Schellenberg, 1838

http://species-id.net/wiki/Paranamixis

#### Generic diagnosis.

(Anamorph males) Antennae relatively long. Eyes with 10 or more ocelli. Maxilliped inner plates generally fused or vestigial; outer plates lacking inner lobes. Coxa 1 greatly reduced, remainder of gnathopod 1 absent, occasionally a small vestige in transformational males.

#### Paranamixis thomasi sp. n.

urn:lsid:zoobank.org:act:92DBD2E7-7C94-40BF-8595-A67059E2231E

http://species-id.net/wiki/Paranamixis_thomasi

Figs 15 16

##### Type material.

Holotype male, 2.6 mm, RUMF-ZC-1690, Sunabe Seawall reef (26°19'25"N, 127°44'43"E), coral rubble, 7–10 m, K.N. White and N.S. White col., 5 October 2010 (KNWOkinawa12D). Paratype male, 2.6 mm, RUMF-ZC-1692, Toguchi Beach patch reef (26°21'47"N, 127°44'12"E), coral rubble, 1–3 m, K.N. White and N.S. White col., 3 February 2011 (KNWOkinawa28B). Paratype female, 2.3 mm, RUMF-ZC-1691, same station data as holotype.

##### Type locality.

Sunabe Seawall, Okinawa, Japan (26°19'25"N, 127°44'43"E).

##### Additional Material Examined.

2 anamorphs, 15 leucomorphs, RUMF-ZC-1693, KNWOkino1B; 2 anamorphs, 19 leucomorphs, NSMT - Cr21835, KNWJap10-9-8A; 1 anamorph, 3 leucomorphs, RUMF-ZC-1694, KNWOkinawa20A; 1 anamorph, 12 leucomorphs, NSMT- Cr21836, KNWOkinawa14G; 1 leucomorph, RUMF-ZC-1700, KNWOkinawa14G; 14 leucomorphs, RUMF-ZC-1695, KNWTokuno4F; 1 anamorph, RUMF-ZC-1696, KNWOkinawa36D; 1 anamorph, RUMF-ZC-1697, KNWOkinawa38D; 1 anamorph, NSMT - Cr21837, KNWIshigaki4J; 1 anamorph, RUMF-ZC-1694, KNWOkinawa47F.

##### Diagnosis (male).

Terminal anamorph head with lateral ridge, anterodistal margin quadrate with cusp. Maxilliped inner plates with small cleft. Coxa 1 anterodistally subtriangular, bi-cuspidate; gnathopod 1 absent. Gnathopod 2 coxa anterior margin expanded with cusp; basis with anterodistal serrate ridge; propodus with 2 mediofacial setal rows, posterior margin serrate; dactylus proximal margin with 1 tubercle and 2 plumose setae. Sub-terminal female Head. Anterior margin truncate. Mandibular palp 1-articulate. Gnathopod 1 carpus terminal ornamentation consisting of 2 serrate blades; propodus palm with 7 sets of 3 setae. Gnathopod 2 propodus palm with 6 major tubercles.

##### Description (Anamorph male).

Head with lateral ridge. Anterior margin oblique, anterodistal margin quadrate with cusp; ventral cephalic keel anterior margin excavate, anteroventral margin subquadrate, ventral margin excavate; eyes with more than 10 ommatidia, round. Antenna 1 0.4 × body length, flagellum 7–articulate, peduncle article 1 width less than 2 × article 2, accessory flagellum absent, aesthetascs present. Antenna 2 0.4 × body length, subequal in length with antenna 1, flagellum 3–articulate. Mouthparts reduced. Maxilliped inner plates with small cleft, bare; outer plate inner margin smooth, reaching 0.1 × palp article 1, bare; palp 4–articulate, article 4 elongate, distally acute.

Pereon. Coxae 1–4 relative widths 1.0: 2.5: 1.6: 2.1. Gnathopod 1 coxa smooth, bare, anterodistal margin produced, subtriangular, bi-cuspidate, distal margin oblique, posterior margin straight, facial setae absent. Gnathopod 1 absent. Gnathopod 2 coxa broader than long, greatly enlarged, smooth, with tiny marginal setae, anterior margin expanded with cusp, anterodistally rounded, distal margin evenly rounded, posterior margin straight, facial setae absent; basis anterodistally expanded, with anterodistal serrate ridge, anterior margin 3 short setae, posterior margin bare; ischium bare; carpus 0.7 × propodus length, curved, distally tapered, anterior margin smooth; propodus with 2 mediofacial setal rows, primary mediofacial setal row above midline, reaching 0.4 × propodus length, secondary mediofacial setal row with 4 setae, with 1 row of submarginal setae, posterior margin serrate, palm convex with 3 major and several minor tubercles; dactylus curved, proximal margin with 1 tubercle and 2 plumose setae, anterior margin distally acute, reaching 0.4 × propodus length. Pereopod 3 coxa length 1.3 × width, anterodistal corner overriding distal face of coxa 2, not extending below it, smooth, with tiny marginal setae, anterior margin expanded, distal margin slightly convex with cusp, posterior margin straight, facial setae absent. Pereopod 4 coxa smooth, with tiny marginal setae, anterior margin produced, distal margin evenly rounded, posterior margin excavate, facial setae absent. Pereopods 5–7 coxae facial setae absent; bases width length ratios 1: 1.4, 1: 1.5, 1: 1.5; posterior margins smooth, setose.

Pleon. Epimera 1–2 with ventral setae, epimeron 3 bare; epimeron 3 posteroventral corner rounded. Uropods 1–3 relative lengths 1.0: 0.7: 0.9. Uropod 1 peduncle subequal in length with inner ramus; outer ramus 0.9 × inner ramus length; inner ramus with 1 robust seta; outer ramus with 4 robust setae. Uropod 2 peduncle 0.8 × inner ramus length; outer ramus 0.6 × inner ramus length; inner ramus with 3 robust setae; outer ramus with 2 robust setae. Uropod 3 peduncle 1.1 × inner ramus length; outer ramus length 0.6 × inner ramus length; inner ramus with 3 robust setae; outer ramus with 2 robust setae. Telson 1.3 × longer than wide, apex rounded.

##### Leucomorph (juvenile and sexually dimorphic characters).

Head. Anterior margin truncate, anterodistal margin subquadrate; ventral cephalic keel anterior margin truncate, anteroventral margin produced, ventral margin straight; eyes with more than 10 ommatidia, round. Antenna 1 0.3 × body length, flagellum 8–articulate. Antenna 2 0.3 × body length, shorter than antenna 1, flagellum 3–articulate. Mandibles lacking molars, palp 1–articulate with 2 distal setae, incisors weakly dentate, left mandible with 5 raker spines, lacinia mobilis large; right mandible with 5 raker spines, lacinia mobilis small. Upper lip asymmetrically lobate, anterior margin setose. Lower lip inner lobes fused, bare; outer lobes with small gape, anterior margins setose. Maxilla 1 palp 1–articulate with 3 distal setae; outer plate with 2 distal robust setae, 6 distal setae. Maxilla 2 inner plate with 2 distal setae; outer plate with 2 distal setae, facial setae present. Maxilliped inner plates fused, distal margin with v-shaped indentation, with short robust setae; outer plate inner margin smooth, reaching 0.2 × palp article 1, with 4 simple marginal setae; palp 4–articulate, article 4 subequal in length with article 3, distally acute.

Pereon. Coxae 1–4 relative widths 1.0: 1.4: 0.9: 1.4. Gnathopod 1 coxa smooth, bare, anterodistal margin rounded, distal margin oblique, posterior margin straight, facial setae absent; basis linear, anterior and posterior margins bare; ischium bare; carpus linear, length 16 × width, proximal margin smooth, with terminal serrate blades and bulbous tip, distal margin with 1 short seta; propodus proximally inflated, palm smooth with 7 sets of 3 proximal setae; dactylus smooth, with large proximal spine, reaching 0.1 × propodus length. Gnathopod 2 coxa equally as long as broad, slightly larger than coxa 3, smooth, with tiny marginal setae, anterior margin expanded, anterodistally subquadrate with cusp, distal margin straight, posterior margin tapered with posteroventral cusp; basis distally expanded, with 4 small anterodistal tubercles; ischium with 1 posterodistal seta; carpus 0.4 × propodus length, straight, distally tapered, anterior margin smooth; propodus with 1 mediofacial setal row displaced to midline, reaching 0.4 × propodus length, with 1 row of submarginal setae, posterior margin smooth, palm subtriangular with 6 major tubercles; dactylus proximal margin smooth, setose, anterior margin distally acute, reaching 0.3 × propodus length. Pereopod 3 coxa length 1.8 × width, smooth, with tiny marginal setae, anterior margin straight, distal margin slightly convex, posterior margin straight, facial setae absent. Pereopod 4 coxa smooth, with tiny marginal setae, anterior margin straight, distal margin evenly rounded, posterior margin tapered, facial setae absent. Pereopods 5–7 coxae facial setae absent; bases width length ratios 1: 1.2, 1: 1.1, 1: 1.2, posterior margins smooth, setose.

Pleon. Epimera 1–2 with ventral setae; epimeron 3 bare, posteroventral corner subquadrate. Uropods 1–3 relative lengths 1.0: 0.7: 1.3. Uropod 1 peduncle 0.6 × inner ramus; outer ramus 0.9 × inner ramus; inner and outer rami each with 3 robust setae. Uropod 2 peduncle and outer ramus 0.6 × inner ramus; inner and outer rami each with 3 robust setae. Uropod 3 peduncle subequal in length with inner ramus; outer ramus 0.6 × inner ramus length; inner ramus with 4 robust setae; outer ramus with 3 robust setae. Telson 1.6 × longer than wide, apex tridentate.

**Figure 15.**
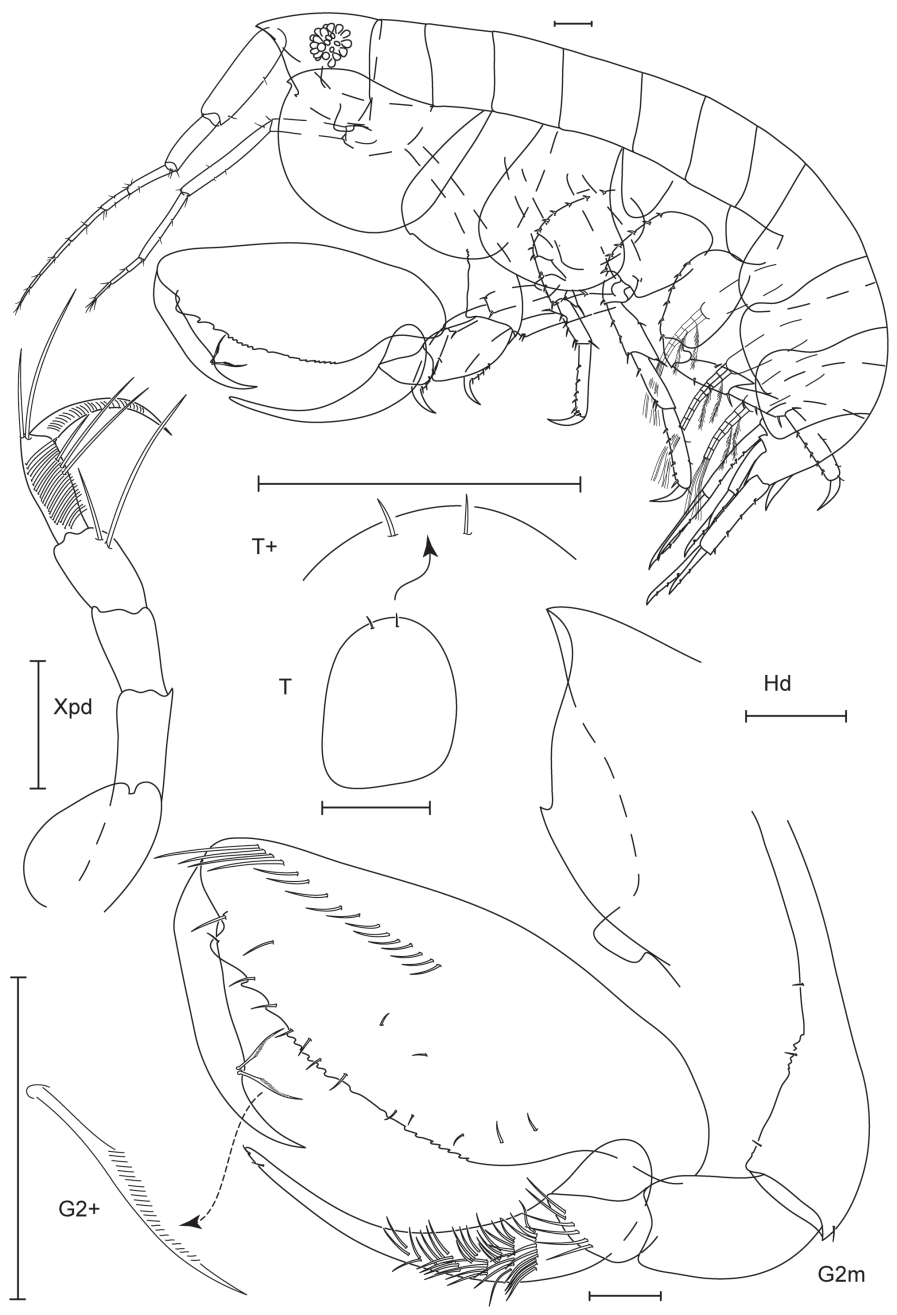
*Paranamixis thomasi* sp. n., holotype male, 2.6 mm, RUMF-ZC-1690.

**Figure 16.**
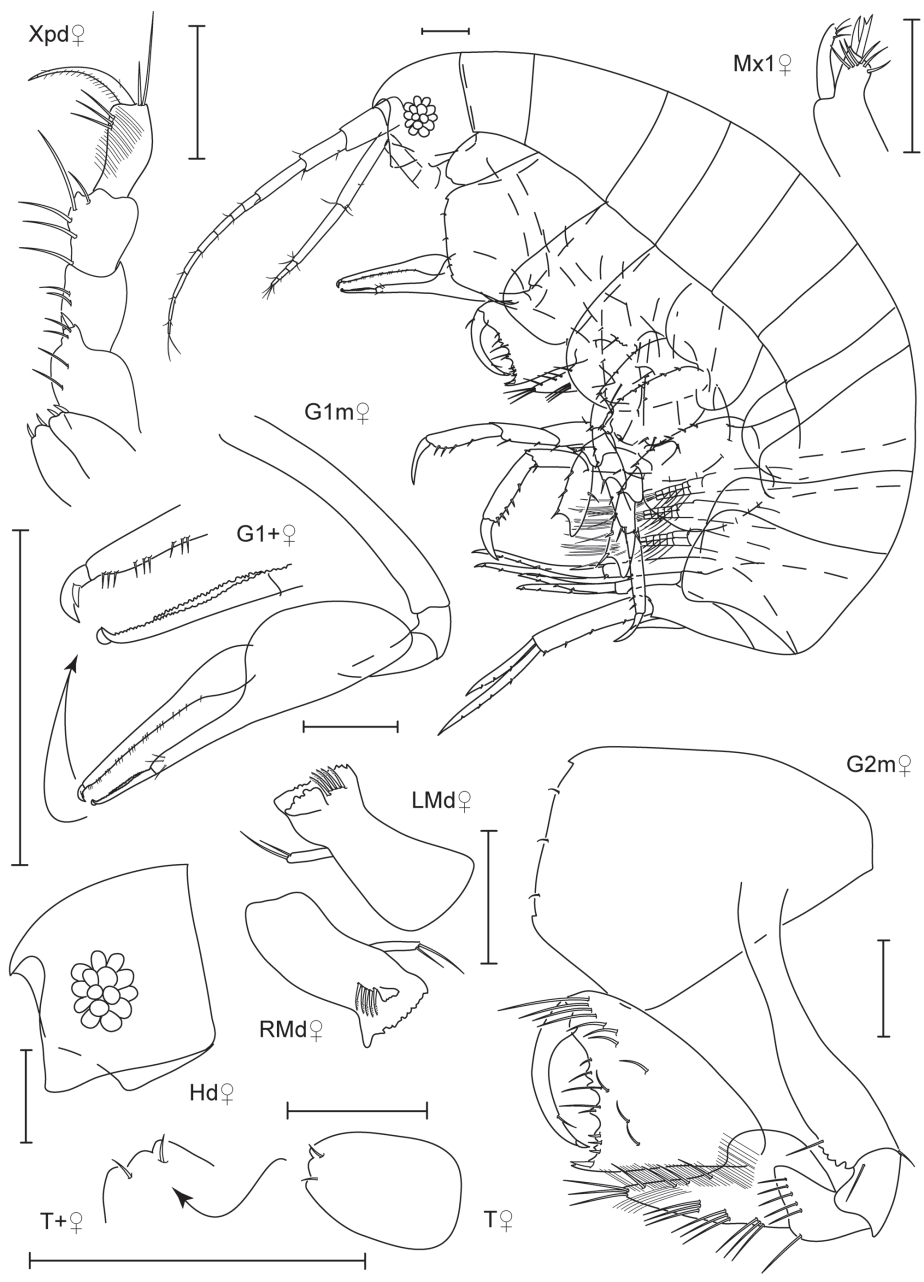
*Paranamixis thomasi* sp. n., paratype female, 2.3 mm, RUMF-ZC-1691.

##### Etymology.

Named for Dr. James Darwin Thomas in recognition of his contribution to amphipod taxonomy, particularly regarding the Leucothoidae. Dr. T has been a mentor and friend for the past 10 years and the first author is very grateful for all his support.

##### Relationships.

*Paranamixis thomasi* is similar to *Paranamixis aberro* in having a cuspate anteroventral head margin and an enlarged gnathopod 2 coxa. *Paranamixis thomasi* differs in having a serrate ridge on the gnathopod basis instead of a large tubercle and in having plumose setae on the dactylus. *Paranamixis thomasi* is similar to *Paranamixis misakiensis* in having the head with a lateral ridge, maxilliped outer plate with a small cleft, an enlarged gnathopod 2 coxa, and a serrate ridge on the gnathopod 2 basis anterior margin. *Paranamixis thomasi* differs from this species in having a single cusp on the anteroventral head margin, a smaller serrate ridge on the gnathopod 2 basis, and smooth gnathopod 2 carpus and dactylus inner margins.

##### Ecology.

In the branchial chamber of the solitary ascidian, *Pyura* sp. (Fig. 18E); and coral rubble.

##### Remarks.

**Figure 17.**
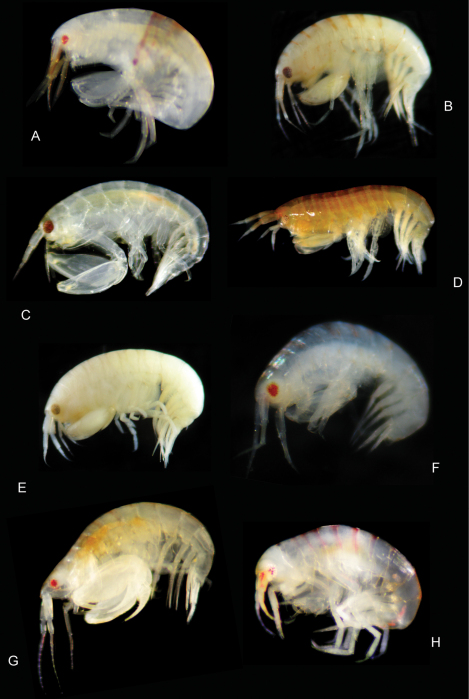
Color plate of new leucothoid amphipod species. **A** *Leucothoe vulgaris* sp. n. **B** *Leucothoe amamiensis* sp. n. **C** *Leucothoe elegans* sp. n. **D** *Leucothoe nathani* sp. n. **E** *Leucothoe obuchii* sp. n. **F** *Leucothoe trulla* sp. n. **G** *Paranamixis thomasi* sp. n. anamorph **H** *Paranamixis thomasi* sp. n. leucomorph

Both anamorphs and leucomorphs are translucent with magenta-pink stripes along the pereonite edges (Figs 17G, H). When collected in an ascidian, one anamorph and two leucomorphs were collected together from one branchial chamber.

##### Distribution.

East China Sea: Okinawa–jima Island, Ishigaki–jima Island, and Iriomote–jima Island (all Okinawa), Tokunoshima Island and Okinoerabu–jima Island (both Kagoshima), Japan.

## Identification Key to ascidian-dwelling Leucothoidae of the Ryukyu Archipelago

**Table:** 

1	Extreme sexual dimorphism; gnathopod 1 absent in post-transformational males; gnathopod 1 carpus with terminal serrate blades in females and pre-transformational males	*Paranamixis thomasi*
–	Minimal sexual dimorphism; gnathopod 1 always present, without terminal ornamentation	2
2	Gnathopod 1 dactylus reaching no more than 0.2 × propodus length; gnathopod 2 carpus distally tapered; pereopods 5–7 bases narrow, oval in shape	3
–	Gnathopod 1 dactylus reaching at least 0.3 × propodus length; gnathopod 2 carpus distally truncate or with subdistal tooth; pereopods 5–7 bases wide, posteriorly tapered	5
3	Anterior head margin truncate; gnathopod 1 propodus palm dentate; gnathopod 2 carpus reaching less than 0.4 × propodus length; pereopods 5–7 bases posteriorly serrate; female gnathopod 1 basis posterior margin with ~15 setae	*Leucothoe elegans*
–	Anterior head margin rounded; gnathopod 1 propodus palm smooth; gnathopod 2 carpus reaching between 0.4 and 0.6 × propodus length; pereopods 5–7 bases posteriorly smooth ; female gnathopod 1 basis posterior margin bare or with 1 seta	4
4	Antenna 1 accessory flagellum absent; maxilla 2 inner plate with serrate robust setae; maxilliped inner plate smooth; male gnathopod 1 basis distally expanded, carpus basally inflated, proximal margin smooth, propodus straight; gnathopod 2 propodus mediofacial setal row with dense tufts of setae	*Leucothoe nathani*
–	Antenna 1 accessory flagellum 1–articulate; maxilla 2 inner plate with simple robust setae; maxilliped outer plate inner margin tuberculate; male gnathopod 1 basis centrally widened, carpus linear, proximal margin dentate, propodus inflated; gnathopod 2 propodus mediofacial setal row sparse single setae	*Leucothoe obuchii*
5	Maxilla 1 palp 1–articulate, margins constricted; mandibular palp article 2 with 5 distal setae; male gnathopod 2 carpus distally spoon-like, propodus mediofacial setal row reaching less than 0.7 × propodus length; telson apex tridentate	*Leucothoe trulla*
–	Maxilla 1 palp 2-articulate; mandibular palp article 2 with 15–17 distal setae; male gnathopod 2 carpus not spoon-like distally or with subdistal tooth, propodus mediofacial setal row reaching greater than 0.7 × propodus length; telson apex strongly pointed or truncated	6
6	Antenna 1 accessory flagellum absent; coxae 1–7 without facial setae; gnathopod 1 basis anterior margin with 6 setae, carpus proximal margin dentate; gnathopod 2 carpus distally truncate; telson apex strongly pointed	*Leucothoe vulgaris*
–	Antenna 1 accessory flagellum 1-articulate; coxae 1–7 with facial setae; gnathopod 1 basis anterior margin with 13–16 setae, carpus proximal margin smooth; gnathopod 2 carpus with large subdistal tooth; telson apex truncated	*Leucothoe amamiensis*

**Figure 18.**
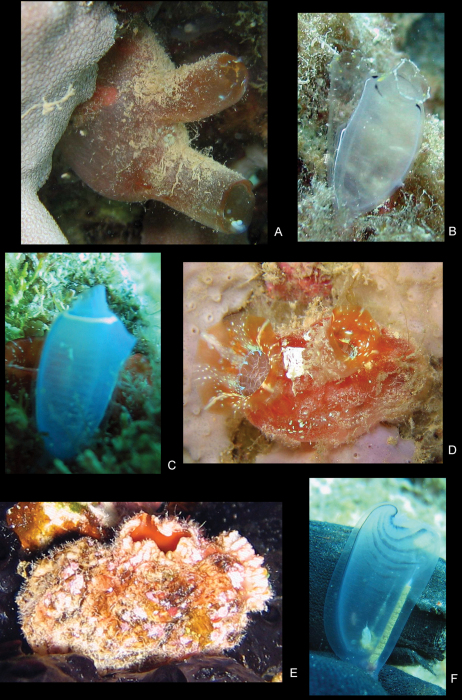
Color plate of ascidian hosts. **A** *Herdmania* of Lahille, 1888 **B** *Rhopalaea* of Phillippi, 1843 **C** *Clavelina* of Savigny, 1816 **D** *Pyura microcosmus* (Savigny, 1816) **E** *Pyura* of Molina, 1782 **F** *Rhopalaea circula* Monniot & Monniot, 2001.

## Discussion

The six *Leucothoe* species described here share the displaced gnathopod 2 propodus mediofacial setal row, a character common to ascidian-dwelling species worldwide. It is likely that this character is an artifact of convergent evolution in species adapting to feeding within similar hosts rather than evidence of relationships between species. *Leucothoe amamiensis*, *Leucothoe elegans*, and *Leucothoe trulla* each have a small accessory flagellum on antenna 1. This character is unusual in leucothoid species and apparently much more common in Pacific species than in Caribbean species. It is particularly interesting because these three species have limited distributions in the Ryukyu Archipelago.

The currently recognized biogeographic boundaries (Hikida and Ota 1997; Ota 1998) do not appear to apply to leucothoid amphipods in the Ryukyu Archipelago despite their restricted distributions. There are some interesting distributional patterns evident in some species, while others, such as *Leucothoe vulgaris* and *Paranamixis thomasi*, are found throughout the entire archipelago. It is possible that these patterns in amphipod distributions are partly attributable to the ephemeral nature of their ascidian hosts. Numbers of ascidian species and individuals in the Ryukyu Archipelago appear to be much higher in the winter months than in the summer. A similar pattern is evident for leucothoid amphipods.

Interesting distributional patterns were observed in *Leucothoe obuchii*, which has been collected from Oura–wan Bay on the northeastern coast of Okinawa–jima Island as well as from two of the northernmost Ryukyu islands, Amami–oshima Island and Yakushima Island; and in *Leucothoe elegans*, whichhas only been collected from Yakushima Island and Shioya Bay on the northeastern coast of Okinawa–jima Island. Both Oura–wan and Shioya bays on the northeastern coast of Okinawa–jima Island are muddy and are ecologically very different from most of the environments on Okinawa–jima Island (Shokita et al. 2002; Naruse et al. 2009). These muddy bays also are very different from the coral reef habitats in the northern Ryukyu Islands that *Leucothoe obuchii* and *Leucothoe elegans* were collected from.

## Supplementary Material

XML Treatment for Leucothoe

XML Treatment for Paranamixis

## Appendix 1

Detailed list of collection stations. (doi: 10.3897/zookeys.163.2003.app) File format: Excel spreadsheet (xls).

**Explanation note:** Appendix I contains station numbers, collection localities, sample descriptions, latitudes, longitudes, dates, depths, and collectors of each collection event.

**Copyright notice:** This dataset is made available under the Open Database License (http://opendatacommons.org/licenses/odbl/1.0/). The Open Database License (ODbL) is a license agreement intended to allow users to freely share, modify, and use this Dataset while maintaining this same freedom for others, provided that the original source and author(s) are credited.
